# Prevalence of Sarcopenia in Patients With COVID-19: A Systematic Review and Meta-Analysis

**DOI:** 10.3389/fnut.2022.925606

**Published:** 2022-07-04

**Authors:** Ying Xu, Jia-wen Xu, Peng You, Bing-Long Wang, Chao Liu, Ching-Wen Chien, Tao-Hsin Tung

**Affiliations:** ^1^Institute for Hospital Management, Tsing Hua University, Shenzhen, China; ^2^Evidence-Based Medicine Centre, Taizhou Hospital of Zhejiang Province Affiliated to Wenzhou Medical University, Linhai, China; ^3^School of Health Policy and Management, Chinese Academy of Medical Sciences and Peking Union Medical College, Beijing, China

**Keywords:** sarcopenia, acute sarcopenia, COVID-19, muscle loss, meta-analysis

## Abstract

**Background:**

It has been speculated that patients with sarcopenia are aggravated by the current novel coronavirus disease 2019 (COVID-19) epidemic. However, there is substantial uncertainty regarding the prevalence of sarcopenia in patients with COVID-19.

**Objectives:**

The purpose of the study was to systematically evaluate the prevalence of sarcopenia in patients with COVID-19, including stratification by gender, study location, study population, study design, and diagnostic criteria.

**Design:**

This is the systematic literature review and meta-analysis.

**Methods:**

An electronic search was performed in MEDLINE/PubMed, Embase, Cochrane Library, and Web of Science and Scopus to identify observational studies reporting a prevalence estimate for sarcopenia in patients with COVID-19. Studies were reviewed in accordance with the Preferred Reporting Items for Systematic reviews and Meta-Analyses (PRISMA) guidelines and a meta-analysis was performed. Risk of bias (RoB) was assessed using the Newcastle–Ottawa Scale (NOS) for cohort studies and Joanna Briggs Institute (JBI) manual for cross-sectional studies, and Stata 14.0 was used to perform meta-analyses.

**Results:**

A total of 4,639 studies were initially identified. After removing the duplicates and applying the selection criteria, we reviewed 151 full-text studies. A total of 21 studies, including 5,407 patients, were eligible for inclusion in this review finally. The prevalence of sarcopenia in patients with COVID-19 in individual studies varied from 0.8 to 90.2%. The pooled prevalence of sarcopenia in COVID-19 was 48.0% (95% confidence interval, CI: 30.8 to 65.1%, *I*^2^ = 99.68%, *p* = 0.000). We did not find any significant differences in the prevalence estimates between gender specificity (OR = 1.34; 95% CI = 0.80–2.26; *p* = 0.001). By sex, the prevalence was 42.5% (95% CI: 31.7 to 53.4%) in men and 35.7% (95% CI: 24.2 to 47.2%) in women. The prevalence estimates significantly varied based on population settings and different diagnostic criteria of sarcopenia. ICU patients (69.7, 95% CI: 51.7 to 85.2%) were more likely to suffer from sarcopenia compared to other population settings.

**Conclusion:**

To our knowledge, this is the first meta-analysis reporting on the prevalence of sarcopenia in patients with COVID-19. Sarcopenia is frequently observed in patients with COVID-19, with varying prevalence across population settings. This study would be useful for clinicians to prompt the increasing awareness of identifying sarcopenia and developing interventions at patients with COVID-19 with high risk of sarcopenia. Further prospective longitudinal studies to define the association of sarcopenia and its prognostic outcomes in COVID-19 survivors are urgently needed to propose the most appropriate treatment strategies during their admission and discharge.

**Systematic Review Registration:**

[www.crd.york.ac.uk/prospero/], identifier [CRD42022300431].

## Introduction

The outbreak of coronavirus disease 2019 (COVID-19), caused by the severe acute respiratory syndrome cornonavirus-2 (SARS-CoV-2), has spread rapidly around the world and impacted most healthcare systems (1). It has been observed that the disease is associated with a wide spectrum of presentations, from seemingly mild asymptomatic disease to severe acute respiratory failure requiring ventilatory support (2), resulting in the damage to multiple organs such as myocardial dysfunction, gastrointestinal symptoms, neurologic illnesses, hepatic injury, and renal injury (3, 4).

Sarcopenia was originally confined to the elderly, defined by the reduced muscle strength with reduced muscle quantity and/or muscle quality (5). It is prevalent up to 15% in healthy older adults (6) and can reach as high as 69% in rehabilitation patients (7). However, emerging evidence suggests that sarcopenia can develop at any age. Other than aging, possible causes, including nutrition, inflammation, vitamin D, critical care admission, and severe illness, are also increasingly recognized as the potential mechanisms contributing to the development of sarcopenia (8, 9). Acute sarcopenia is an emerging condition of acute muscle insufficiency, defined by the European Working Group on Sarcopenia in Older People 2 (EWGSOP2) as incident sarcopenia within 6 months, normally following a stressor event (5). No matter what type of sarcopenia, it is associated with poor health outcomes such as falls (10), cognitive impairment (11), depression (12), fractures (13), and increased mortality (14). In addition, previous studies have demonstrated that the presence of sarcopenia may be a predictor of treatment outcomes in patients with acute or chronic illness and those undergoing surgery (15–19).

The relationship between sarcopenia and COVID-19 has received substantial interest in the current literature. Hospitalization has been confirmed to be associated with acute changes in sarcopenia status in older people (20, 21). Studies of hospitalized patients have described biochemical evidence of muscle damage (22) during the pandemic, and it has been speculated that patients with COVID-19 are at increased risk of acute sarcopenia (23, 24), which is characterized by low skeletal muscle mass (LSMM) and reduced strength. Observation from numerous studies has shown that LSMM is predicted to have dismal prognoses amid the COVID-19, associated with higher in-hospital mortality (25–28), extubation failure (25), longer hospital length of stay (26, 29), longer intensive care unit (ICU) length of stay (25, 29), higher ICU admission (30), and severe condition (31–33). Previous studies have also found a significant association between reduced muscle strength and COVID-19 severity (31, 33). Additionally, according to a published meta-analysis, there is evidence that skeletal muscle quality, rather than mass, is associated with COVID-19 severity (34).

Though studies which addressed the potential mechanisms and management between acute sarcopenia and COVID-19 have already existed (8, 35, 36), few studies have described the prevalence of sarcopenia among patients with COVID-19, based on different population settings and screening tools. Determining the prevalence of sarcopenia in patients with COVID-19 is critical to develop diagnoses and treatments for the condition. Therefore, the aim of this systematic review and meta-analysis was to establish summary estimates for the prevalence of sarcopenia in patients with COVID-19, including stratification by gender, study location, study population, study design, and diagnostic criteria.

## Methods

### Study Registration

The systematic review was performed in accordance with Preferred Reporting Items for Systematic Reviews and Meta-Analyses (PRISMA) guidelines, and the protocol of this systematic review was registered in the PROSPERO under the number CRD42022300431.

### Literature Search

A systematic search was conducted in MEDLINE/PubMed, Embase, Cochrane Library, and Web of Science and Scopus from inception date until 19 May 2022. The search strategy consisted of a combination of appropriate Mesh term and other key terms, which included “coronavirus infections,” “coronavirus,” “COVID-19,” “SARS-CoV-2,” “severe acute respiratory syndrome,” “2019-nCoV,” “sarcopenia,” “muscular atrophy,” “muscle weakness,” “muscle loss,” “muscle depletion,” “muscle reduction,” “muscle wasting,” “loss of muscle,” “low muscle mass,” and “body composition.” We further hand-searched the reference section of included publications to identify the potential articles missed by the initial search. The full search strategy can be found in Supplementary Table 3.

### Study Selection

The whole studies of literature were first assessed for eligibility through title and abstract screening by two independent reviewers (Y.X. and J-W.X). Then, the full text of potentially relevant studies was further evaluated according to the PECOS (population, exposure, comparison/comparator, outcome, and study type) criteria (37): (i) population: general population or hospitalized population; (ii) exposure: the exposures of interest are infection with COVID-19; (iii) comparison/comparator: the comparator will be healthy population that without COVID-19, (iv) outcome: the outcome of interest is diagnosis of sarcopenia; and (v) study type: observational studies (cohort studies, case–control, and cross-sectional studies). Studies were excluded if: (1) wrong publication type (letters to the editor, review, editorials), (2) unqualified study design (e.g., animal studies, case report, randomized-control study), (3) without defined sarcopenia, (4) not report the prevalence of sarcopenia, and (5) not published in English. Disagreements during the screening process were resolved through consensus from a third senior investigator (T.H.T).

### Quality Assessment

The bias risk assessment of cohort studies was assessed by two independent reviewers (Y.X. and J-W.X.) using the Newcastle–Ottawa Scale (NOS). The NOS evaluates the quality of a study through three criteria: (1) selection, (2) comparability, and (3) outcome (38). High-quality articles were defined as ≥7 stars (39). Cross-sectional studies were critically appraised by two independent reviewers (Y.X. and J-W.X.) using the Joanna Briggs Institute (JBI) Critical Appraisal Checklists. Disagreements were resolved by a third author (T.H.T) to review the data.

### Data Extraction

There were two independent authors (Y.X. and J-W.X.) who reviewed the 8 included studies in the data extraction process, and a third author (T.H.T) was consulted to resolve discrepancy. The following items of studies were extracted: main characteristics (author, publication year), study characteristics (country, study design, study setting, sample size, and clinical outcome), patients characteristics (gender, range of age), and diagnostic criteria of sarcopenia (assessment tools, the investigated level/muscles, sarcopenia parameters, and cutoff used).

### Statistical Analysis

The prevalence of sarcopenia with 95% confidence intervals (CIs) was pooled using a meta-analysis of single proportions. If a study included the event of sarcopenia, both exposed group and non-exposed group, odds ratios (OR) for sarcopenia were calculated as well. Heterogeneity was assessed with the I^2^ statistics and significance with the Cochrane Q statistic. The Cochrane Q statistic, with a significance level of *p* < 0.10, was used to assess the presence of heterogeneity. The I^2^ statistics was further used to quantify the magnitude of the heterogeneity, with values of <25, 25–75, and >75% indicating low, moderate, and high heterogeneity, respectively, as recommended by the Cochrane Handbook (40). Given *p* ≤ 0.10, *I*^2^ ≥ 50%, we used the random-effects model (REM); otherwise, a fixed-effects model (FEM) would be adopted. Furthermore, subgroup analyses were performed to determine the distribution of sarcopenia by sex, study location, study population, study design, and diagnostic criteria of sarcopenia. We also conducted the sensitivity analysis to assess the effect of every study on the synthesized estimate of the prevalence. Publication bias was tested visually using the funnel plots and statistically using the Egger’s test, and *p* < 0.05 was considered to indicate a statistically significant publication bias. All statistical analyses were performed using All meta-analyses were performed using STATA version 14.0 (Stata Corporation, College Station, TX, United States).

## Results

### Search Results

**FIGURE 1:**
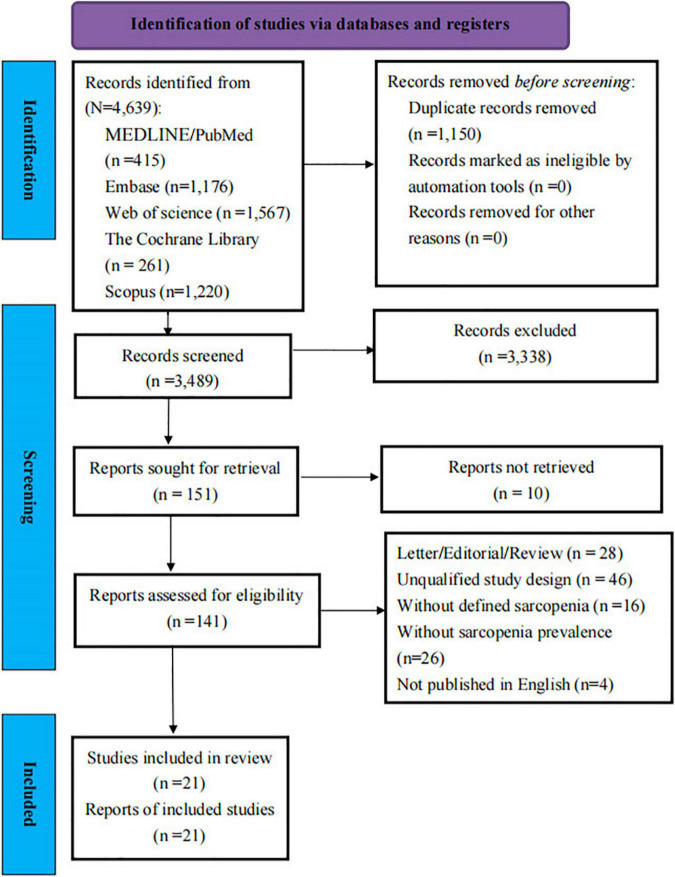
PRISMA (2020) diagram of study screening and selection.

The search strategy identified 4,639 articles through the electronic database searches. A total of 1,150 duplicate articles were removed. Of these, 3,338 articles were excluded after screening the titles and abstracts, leaving 151 articles for full-text review. Finally, 21 articles (*n* = 5,407 patients with COVID-19) (25–27, 29–33, 41–53) were included in the systematic review and meta-analysis finally. Figure 1 presents the PRISMA diagram for the study selection and reasons for exclusion.

### Characteristics of Included Studies

Table 1 shows the characteristics of the included studies. The 21 included studies comprised of 5,407 patients with COVID-19 with the mean age ranging from 44.5 to 86.1 years. Most studies (14/21) included individuals from Europe [2 from Spain (41, 44), 4 from Italy (25, 30, 50, 51), 2 from France (43, 49), 3 from the United Kingdom (27, 48, 53), 1 from Germany (52), and 1 from Netherlands (45)], and the rest of included studies (7/21) included individuals from Asia [3 from China (31, 33, 46), 2 from Turkey (26, 32), and 1 from South Korea (42)] and North America [2 from Mexico (29, 47)]. Only three included studies had a cross-sectional design (32, 44, 50), and the remaining eighteen studies were observational cohort studies (25–27, 29–31, 33, 41–43, 45–49, 51–53). A total of eleven included studies (*n* = 1,603 patients with COVID-19) described the specific sarcopenia events between male and female patients with COVID-19 (26, 29–31, 42, 43, 47, 49–52). Regarding the target population, most studies (20/21) recruited hospitalized patients (18 of 20 studies provided available information about patients’ hospitalization status), and the remaining one study recruited general population. With respect to the assessment tool of sarcopenia: (1) A number of five studies measured sarcopenia through chest CT-scan: four studies with 1,024 patients analyzed muscle at the level of the twelfth thoracic (T12) (30, 33, 42, 47) and one study with 130 patients analyzed the pectoralis musculature (26); (2) a number of six studies measured sarcopenia through abdominal CT-scan: five studies with 430 patients analyzed muscle at the third lumbar (L3) vertebra (27, 29, 46, 52, 53) and one study with 81 patients analyzed muscle at the L1, L2, or L3 level (25); (3) a number of four studies with 846 patients measured sarcopenia through the strength, assistance in walking, rise from a chair, climb stairs, falls history questionnaire (SARC-F) scale (31, 41, 44, 45); (4) then, one study with 23 patients measure sarcopenia through the medical research council (MRC) scale (43); (5)a number of two studies with 567 patients measured sarcopenia through dynamometer (32, 50); (6) A total of two studies with 2,167 patients measured sarcopenia through bioelectrical impedance analysis (BIA) (48, 51); (7) and one study with 139 patients measured sarcopenia using dynamometer and dual-energy X-ray absorptiometry (DXA) (49). Sarcopenia definitions and their parameters with cutoff values used in included articles are shown in Table 2.

**TABLE 1:** Main characteristics of the studies included in the meta-analysis.

No	Study	Study design	COVID-19 testing	Hospital Setting	Sample size	Subjects F/M^a^	Male (%)	Age (years)^b^	BMI (kg/m2)^b^	Prevalence (%)	Time of assessment	Clinical outcome
1	Ufuk et al. (26) 2020 Turkey	OCS	RT-PCR	NR	130	ICU or COVID-19 nursing wards patients. All: 130 (54/76) Sarcopenia: 44 (19/25) Non-sarcopenia: 86 (35/51)	58.46	48 (36–64)	26.9 (17.1–36.5)	33.85%	NR	Intubation, prolonged hospital stay, and death
2	Yang et al. (46) 2020 China	OCS	NR	Tongji Hospital in Wuhan, China	143	ICU or COVID-19 nursing wards patients. All: 143 (NR/NR) Sarcopenia: 71 (NR/NR) Non-sarcopenia: 72 (NR/NR)	48.95	66 (56–73.5)	23.4 (21.9–25.3)	49.65%	NR	Critical illness
3	Cuerda et al. (41) 2021 Spain	OCS	NR	16 public hospitals of the Community of Madrid	176	ICU patients. All: 176 (50/126) Sarcopenia: 153 (NR/NR) Non-sarcopenia: 23 (NR/NR)	71.59	60.3 ± 10.5	NR	86.93%	At hospital discharge	Nutritional and functional status and the quality of life of patients admitted in ICU
4	Damanti et al. (25) 2021 Italy	OCS	RT-PCR	A tertiary hospital	81	ICU patients. All: 81 (10/71) Sarcopenia: 53 (NR/NR) Non-sarcopenia: 28 (NR/NR)	87.65	59.3 ± 11.91	28.3 ± 4.74	65.43%	NR	Extubation success, length of ICU stay and hospital mortality
5	Giraudo et al. (30) 2021 Italy	OCS	RT-PCR	A tertiary center	150	ICU or COVID-19 nursing wards patients. All: 150 (46/104) Sarcopenia: 43 (16/27) Non-sarcopenia: 107 (30/77)	69.33	61.3 ± 15	NR	28.67%	After hospital admission	ICU admission
6	Kim et al. (42) 2021 South Korea	OCS	RT-PCR	Daegu Catholic University Medical Center	121	COVID-19 nursing wards patients. All: 121 (77/44) Sarcopenia: 29 (18/11) Non-sarcopenia: 92 (59/33)	36.36	62.0 (49.0–75.0)	NR	23.97%	At the time of admission	LOS and mortality
7	Ma et al. (31) 2021 China	OCS	RT-PCR	General Hospital	114	COVID-19 nursing wards patients. All: 114 (57/57) Sarcopenia: 38 (19/19) Non-sarcopenia: 76 (38/38)	50.88	69.52 ± 7.25	23.46 ± 3.18	33.33%	Within 24 h of admission	Development of severe disease
8	Medrinal et al. (43) 2021 France	OCS	NR	ICU tertiary Hospital Settings	23	ICU patients. All: 23 (6/17) Sarcopenia: 16 (3/13) Non-sarcopenia: 7 (3/4)	73.91	64.6 ± 9.6	29.1 ± 3.5	69.57%	NR	MV, prone position and catecholamine
9	Riesgo et al. (44) 2021 Spain	CSS	RT-PCR	Reference hospital	337	COVID-19 nursing wards patients. All: 337 (170/167) Sarcopenia: 304 (NR/NR) Non-sarcopenia: 33 (NR/NR)	49.55	86.1 ± 8.7	23.8 ± 2.8	90.21%	During the first 24 h of hospitalization	Mortality
10	Wierdsma et al. (45) 2021 Netherlands	OCS	NR	3 Dutch hospitals	219	ICU or COVID-19 nursing wards patients. All: 219 (NR/NR) Sarcopenia: 159 (NR/NR) Non-sarcopenia: 60 (NR/NR)	NR	NR	NR	72.60%	During hospital admission and after discharge	Nutritional status
11	Kara O et al. (32) 2021 Turkey	CSS	PCR	270-bed university-affiliated hospital	312	COVID-19 nursing wards patients. All: 312 (140/172) Sarcopenia: 40 (NR/NR) Non-sarcopenia: 272 (NR/NR)	55.13	46.1 ± 14.8	NR	12.82%	At the time of admission	Disease severity
12	McGovern J et al. (27) 2021 United Kingdom	OCS	PCR test or chest X-ray or CT thorax	Glasgow Royal Infirmary	63	ICU or COVID-19 nursing wards patients. All: 63 (33/30) Sarcopenia: 39 (NR/NR) Non-sarcopenia: 24 (NR/NR)	47.62	NA^c^	NA^d^	61.90%	NR	ITU admission and 30-d mortality
13	Moctezuma-Velazquez P et al. (47) 2021 Mexico	OCS	RT-PCR	A tertiary care center	519	ICU or COVID-19 nursing wards patients. All: 519 (187/332) Sarcopenia: 115 (21/94) Non-sarcopenia: 404 (166/238)	63.97	51 (42–61)	29.7 (26.7–33.4)	22.16%	At the time of admission	In-hospital mortality, need of IMV, and/or ICU admission
14	Yi X et al. (33) 2021 China	OCS	RT-PCR	Six designated hospitals for treating patients with COVID-19	234	NR All: 234 (101/133) Sarcopenia: 78 (NR/NR) Non-sarcopenia: 156 (NR/NR)	56.84	44.5 (2.0–81.0)	NR	33.33%	At the time of admission	Risk of transition to severe COVID-19 infection
15	Gobbi et al. (51) 2021 Italy	OCS	RT-PCR	Rehabilitation Unit from several COVID hospitals	34	ICU or COVID-19 nursing wards patients. All: 34 (18/16) Sarcopenia: 20 (9/11) Non-sarcopenia: 14 (9/5)	47.06	NA^e^	NA^f^	58.82%	At the time of admission	Respiratory, body composition, muscle strength and functional parameters considered
16	Wilkinson et al. (48) 2021 United Kingdom	OCS	NR	General population, recruited into United Kingdom Biobank study	2133	NA All: 2133 (NR/NR) Sarcopenia: 16 (NR/NR) Non-sarcopenia: 2117 (NR/NR)	NR	NR	NR	0.75%	NR	Disease severity
17	Osuna-Padilla et al. (29) 2022 Mexico	OCS	RT-PCR and suggestive tomographic findings	The ICU of the National Institute of Respiratory Diseases	86	ICU patients. All: 86 (23/63) Sarcopenia: 41 (15/26) Non-sarcopenia: 45 (8/37)	73.26	48.6 ± 12.9	29.2 ± 5.5	47.67%	At the time of admission	ICU and LOS, tracheostomy, days on MV, and in-hospital mortality
18	Molwitz et al. (52) 2022 Germany	OCS	RT-PCR	University Medical Hospital	32	ICU patients. All: 32 (12/20) Sarcopenia: 24 (6/18) Non-sarcopenia: 8 (6/2)	62.50	64.4 ± 11.4	27.3 ± 6.2	75.00%	NR	LOS, IMV, and time to death
19	Levy et al. (49) 2022 France	OCS	RT- PCR or radiological findings	Strasbourg University Hospital	139	ICU or COVID-19 nursing wards patients. All: 139 (44/95) Sarcopenia: 22 (5/17) Non-sarcopenia: 117 (39/78)	68.35	62 (29–82)	29 (21–44)	15.83%	Three months after discharge	Long term evolution of malnutrition and sarcopenia
20	Damanti et al. (50) 2022 Italy	CSS	NR	San Raffaele University Hospital	255	ICU or COVID-19 nursing wards patients. All: 255 (103/152) Sarcopenia: 121 (53/68) Non-sarcopenia: 134 (50/84)	59.61	67 (56–75)	28 (24.87–31.01)	47.45%	One month after hospital discharge	Muscle ultrasound characteristics (thickness, stiffness and pennation angle)
21	McGovern J et al. (53) 2022 United Kingdom	OCS	PCR test or chest X-ray or CT thorax	Glasgow Royal Infirmary	106	NR All: 106 (50/56) Sarcopenia: 85 (NR/NR) Non-sarcopenia: 21 (NR/NR)	52.83	NA^g^	NA^h^	80.19%	NR	Systemic inflammation

*^a^F/M, female/male.*

*^b^Data are presented as mean ± SD or median (IQR) unless otherwise specified.*

*^c^The author indicated that age categories were grouped to <70 year (n = 21) or ≥70 years (n = 42).*

*^d^The author indicated that BMI categories were grouped to ≥25 (n = 31) or ≥15 (n = 15).*

*^e^The author reported that the mean age of patients with sarcopenia was 71.5 ± 17.0, and the mean age of patients with non-sarcopenia was 68.0 ± 16.5.*

*^f^The author reported that the mean BMI of patients with sarcopenia was 21.0 ± 4.2, and the mean age of patients with non-sarcopenia was 27.3 ± 9.0.*

*^g^The author indicated that age categories were grouped to <70 year (n = 35) or ≥70 years (n = 71).*

*^h^The author indicated that BMI categories were grouped to ≤25 (n = 48) or >25 (n = 58).*

*OCS, observational cohort study; CSS, cross-sectional study; COVID-19, coronavirus disease 2019; RT-PCR, reverse transcription polymerase chain reaction; PCR, polymerase chain reaction; CT, computed tomography; ICU, intensive care unit; BMI, body mass index; LOS, length of stay; MV, mechanic ventilation; ITU, intensive therapy units; IMV, invasive mechanical ventilation; NA, not applicable; NR, not reported.*

**TABLE 2:** Sarcopenia diagnosis and their parameters with cutoff values used in the included studies.

No	First author, year	Sarcopenia assessment tool	The investigated level/muscles	Sarcopenia parameters	Cutoff used
1	Ufuk et al. (26) 2020	Chest CT-scan	Pectoralis muscle	Pectoralis muscle index (PMI)	First tertile of PMI values, for men 12.73 cm^2^/m^2^ and for women 9 cm^2^/m^2^
2	Yang et al. (46) 2020	Abdominal CT-scan	Every muscle on L3 level	Skeletal muscle area (SMA)	Sex-specified median value as threshold
3	Cuerda et al. (41) 2021	SARC-F	NA	SARC-F scale which consist of five component: strength; assistance walking; rise from a chair; climb stairs; and falls (score 0–10)	Total score ≥ 4
4	Damanti et al. (25) 2021	Abdominal CT-scan	Every muscle on L1, L2 or L3 level; L3 were preferentially chosen when available	Skeletal muscle index (SMI)	According to vertebra levels and literature data
5	Giraudo et al. (30) 2021	Chest CT-scan	The right paravertebral muscle at T12 level	The mean Hounsfield Unit (Hu) value	Hounsfield unit (Hu) values < 30
6	Kim et al. (42) 2021	Chest CT-scan	Every muscle on T12 level	Skeletal muscle index (SMI)	Men: 24 cm^2^/m^2^ Women: 20 cm^2^/m^2^
7	Ma et al. (31) 2021	SARC-F	NA	SARC-F scale which consist of five component: strength; assistance walking; rise from a chair; climb stairs; and falls (score 0–10)	Total score ≥ 4
8	Medrinal et al. (43) 2021	MRC scale	NA	The MRC scale of muscle strength uses a score of 0 to 5 to grade the power of a particular muscle group in relation to the movement of a single joint.	Total score ≤ 48/60
9	Riesgo et al. (44) 2021	SARC-F	NA	SARC-F scale which consist of five component: strength; assistance walking; rise from a chair; climb stairs; and falls (score 0–10)	Total score ≥ 4
10	Wierdsma et al. (45) 2021	SARC-F	NA	SARC-F scale which consist of five component: strength; assistance walking; rise from a chair; climb stairs; and falls (score 0–10)	Total score ≥ 4
11	Kara O et al. (32) 2021	Electronic Smedley hand dynamometer	NA	Handgrip strength (in kg)	Two standard deviations below the gender-specific peak mean value of the healthy young adults (i.e., <32 kg in males and <19 kg in females)
12	McGovern J et al. (27) 2021	Abdominal CT-scan	Every muscle on L3 level	Body mass index (BMI) and Skeletal muscle index (SMI)	Men: BMI < 25 kg/m^2^ and SMI < 43 cm^2^/m^2^, or BMI 25 and SMI < 53 cm^2^/m^2^ Women: BMI < 25 and SMI < 41 cm^2^/m^2^, or BMI 25 and SMI < 41 cm^2^/m^2^
13	Moctezuma-Velazquez P et al. (47) 2021	Chest CT-scan	Every muscle on T12 level	Skeletal muscle index (SMI)	Men: < 42.6 cm^2^/m^2^ Women: < 30.6 cm^2^/m^2^
14	Yi X et al. (33) 2021	Chest CT-scan	Every muscle at T12 level	Skeletal muscle index (SMI)	ALM index (ALM/height^2^) <7.26 kg/m^2^ for men and <5.45 kg/m^2^ for women as per EWGSOP2 criteria
15	Gobbi et al. (51) 2021	Bioelectrical Impedance Analysis (BIA)	NA	Appendicular Skeletal Muscle Mass (ASM)	ASM < 20 (kg) for males and ASM < 15 (kg) for females according to EWGSOP2 criteria
16	Wilkinson et al. (48) 2021	Bioelectrical impedance analysis (BIA)	NA	Appendicular lean mass (ALM)/height2 index or ALM/body mass index (BMI)	ALM index (ALM/height^2^) <7.26 kg/m^2^ for men and <5.45 kg/m^2^ for women as per EWGSOP2 criteria; or ALM/body mass index (BMI) <0.789 in men and <0.512 in women as per Foundation for the National Institutes of Health Sarcopenia Project criteria
17	Osuna-Padilla et al. (29) 2022	Abdominal CT-scan	Every muscle on L3 level	Skeletal muscle index (SMI)	BMI < 30 kg/m^2^: SMI ≤ 52.3 cm^2^/m^2^ for men and ≤38.6 cm^2^/m^2^ for women. BMI ≥ 30 kg/m^2^: SMI ≤ 54.3 cm^2^/m^2^for men and ≤46.6 cm^2^/m^2^ for women
18	Molwitz et al. (52) 2022	Abdominal CT-scan	Every muscle on L3 level	Skeletal muscle index (SMI)	Men: <52.4 cm^2^/m^2^ Women: <38.5 cm^2^/m^2^
19	Levy et al. (49) 2022	Hydraulic Hand Dynamometer and dual-energy X-ray absorptiometry (DXA)	NA	Handgrip strength (HGS) and appendicular skeletal muscle mass (ALM)	(1) HGS Men: <27 kg Women: <16 kg. (2) ALM Men: <7.0 kg/m^2^ - Women: <5.5 kg/m^2^
20	Damanti et al. (50) 2022	Dynamometer	NA	Handgrip strength (HGS)	Men: <27 kg Women: <16 kg
21	McGovern J et al. (53) 2022	Abdominal CT-scan	Every muscle on L3 level	Skeletal muscle index (SMI)	According to literature data

*CT, computed tomography; SARC-F, strength, assistance in walking, rise from a chair, climb stairs, falls history questionnaire; MRC, Medical Research Council; EWGSOP2, the European Working Group on Sarcopenia in Older People 2.*

### Quality Assessment

Quality assessment of all observational cohort studies was assessed by NOS. Supplementary Table 4 shows the total NOS score and individual question scores for each included study. The rate of all included studies ranged from 6 to 9. A total of fifteen of eighteen included cohort studies (25–27, 29–31, 33, 41, 42, 46–49, 52, 53) were rated as high quality with NOS score ≥7. Meanwhile, we used JBI Critical Appraisal Checklist for the evaluation of three cross-sectional studies (32, 44, 50), which indicated all included studies had good quality (Supplementary Table 5).

### Prevalence of Sarcopenia Among Patients With COVID-19

**FIGURE 2:**
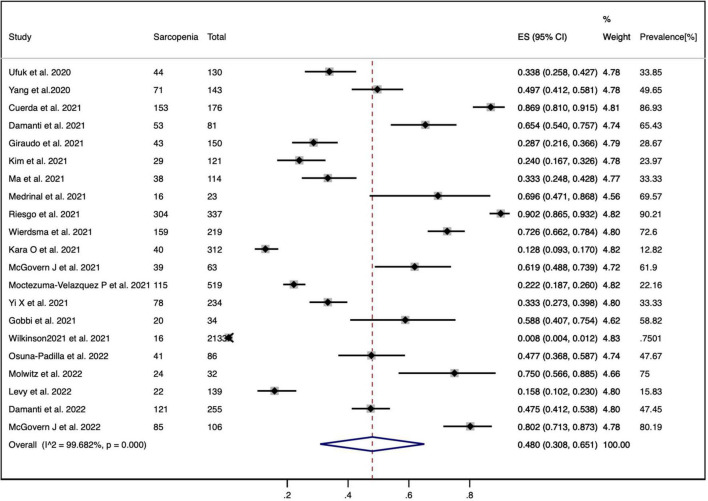
The pooled prevalence of sarcopenia in COVID-19 patients.

The random-effects model was used for the meta-analysis of the pooled prevalence of sarcopenia among patients with COVID-19 as the highly heterogeneity (*p* = 0.000, *I*^2^ = 99.68%). The prevalence of sarcopenia in the included studies varied, ranging from 0.8 to 90.21%. Figure 2 shows that the pooled prevalence of sarcopenia among patients with COVID-19 was 48.0% (95% CI: 30.8 to 65.1%).

### Subgroup Meta-Analyses of the Prevalence of Sarcopenia in Patients With COVID-19

To identify potential effect modifiers on the pooled prevalence of sarcopenia, we performed a subgroup analysis of sex, study location, different target population, study design, and diagnostic criteria of sarcopenia.

#### Sex

**FIGURE 3:**
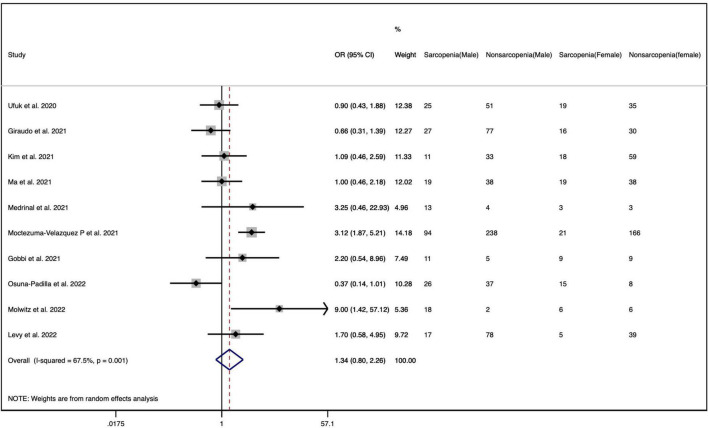
The pooled odds ratios of the association between gender and COVID-19-related sarcopenia.

There are 11 studies reporting the sex-stratified data on sarcopenia in patients with COVID-19 (26, 29–31, 42, 43, 47, 49–52). Table 3 presents that the pooled prevalence of sarcopenia in men with COVID-19 was 42.5% (95% CI: 31.7 to 53.4%), showing a high heterogeneity (*I*^2^ = 92.41%, *p* = 0.000), and the pooled prevalence in women was 35.7% (95% CI: 24.2 to 47.2%), showing a high heterogeneity (*I*^2^ = 90.28%, *p* = 0.000) (Supplementary Figure 1). Furthermore, the OR of the association between gender and COVID-19-related sarcopenia was calculated, as derived from ten observational cohort studies among these eleven retrieved studies providing sex-stratified data (26, 29–31, 42, 43, 47, 49, 51, 52). Figure 3 demonstrates no significant association between gender and COVID-19-related sarcopenia (OR = 1.341; 95% CI: 0.796–2.258; *p* = 0.270).

**TABLE 3:** Subgroup analysis of the prevalence of sarcopenia.

Subgroup	No. of Studies	Events	Total	Pooled prevalence (%)	95% CI	*I*^2^ (%)	*p*-value
**Sex**							
male	11	329	976	42.5	31.7–53.4	92.41%	0.000
female	11	184	627	35.7	24.2–47.2	90.28%	0.000
**Regions**							
Europe	13	1055	3748	57.1	26.6–84.9	99.62%	0.000
Asia	6	300	1054	30.4	19.6–42.4	93.88%	0.000
North America	2	156	605	25.4	21.9–28.9	95.00%	0.000
**Countries**							
Turkey	2	84	442	18.2	14.7–21.9	81.20%	0.000
China	3	187	491	38.6	28.4–49.3	82.10%	0.004
Spain	2	457	513	89.1	86.3–91.7	15.40%	0.277
Italy	4	237	520	49.2	33.6–65.0	91.17%	0.000
South Korea	1	29	121	24.0	16.7–32.6	−	−
France	2	38	162	21.8	15.6–28.7	96.50%	0.000
Netherlands	1	159	219	72.6	66.2–78.4	−	−
United Kingdom	3	140	2302	41.2	0–98.9	99.60%	0.000
Mexico	2	156	605	25.4	21.9–28.9	95.00%	0.000
Germany	1	24	32	75.0	56.6–88.5	−	−
**Study population**							
ICU	5	287	398	69.7	51.7–85.2	91.58%	0.000
ICU or COVID-19 nursing wards	8	513	1397	42.0	26.5–58.3	97.10%	0.000
COVID-19 nursing wards	2	67	235	28.4	22.8–34.4	60.60%	0.111
**Study design**							
OCS	18	1046	4503	46.4	27.6–65.7	99.28%	0.000
CSS	3	465	904	50.8	7.1–93.7	99.80%	0.000
**Assessment tools**							
Chest CT-scan	5	309	1154	28.0	22.8–33.5	72.82%	0.005
Abdominal CT-scan	6	313	511	63.3	51.4–74.4	85.98%	0.000
SARC-F	4	654	846	73	49.3–91.3	98.00%	0.000
MRC	1	16	23	69.6	47.1–86.8	−	−
Dynamometer	2	161	567	26.7	23.2–30.5	98.90%	0.000
BIA	2	36	2167	0.4	0.1–0.8	97.70%	0.000
Dynamometer and DXA	1	22	139	15.8	10.2–23.0	−	−
**Parameters**							
PMI	1	44	130	33.8	25.8–42.7	−	−
SMA	1	71	143	49.7	41.2–58.1	−	−
SARC-F score	4	654	846	73.0	49.3–91.3	98.00%	0.000
SMI	7	425	1179	49.0	31.3–66.9	97.03%	0.000
Hu value	1	43	150	28.7	21.6–36.6	−	−
MRC score	1	16	23	69.6	47.1–86.8	−	−
HGS	2	161	567	26.7	23.2–30.5	98.90%	0.000
BMI and SMI	1	39	63	61.9	48.8–73.9	−	−
ASM	1	20	34	58.8	40.7–75.4	−	−
ALM and BMI	1	16	2133	0.8	0.4–1.2	−	−
HGS and ALM	1	22	139	15.8	10.2–23.0	−	−
**Total**	21	1511	5407	48.0	30.8–65.1	99.68%	0.000

*ICU, intensive care unit; COVID, coronavirus disease 2019; OCS, observational cohort study; CSS, cross-sectional study; CT, computed tomography; SARC-F, strength, assistance in walking, rise from a chair, climb stairs, falls history questionnaire; MRC, Medical Research Council; BIA, bioelectrical impedance analysis; DXA, dual-energy X-ray absorptiometry; PMI, pectoralis muscle index; SMA, skeletal muscle area; SMI, skeletal muscle index; Hu value, Hounsfield unit value; BMI, body mass index; ASM, appendicular skeletal muscle mass; ALM, appendicular lean mass; HGS, handgrip strength; CI, confidential interval.*

#### Study Location

Among the 2 studies conducted in Spain (41, 44), the pooled prevalence of sarcopenia was 89.1% (95% CI: 86.3 to 91.7%, *k* = 2, *I*^2^ = 15.4%, *p* = 0.277). The prevalence in the 4 Italy studies (25, 30, 50, 51) was 49.2% (95% CI: 33.6 to 65.0%, *k* = 4, *I*^2^ = 91.17%, *p* = 0.000). Of the three studies conducted in the United Kingdom (27, 48, 53), the pooled prevalence of sarcopenia was 41.2% (95% CI: 0 to 98.9%, *k* = 3, *I*^2^ = 99.60%, *p* = 0.000). Among the 2 studies conducted in France (43, 49), the pooled prevalence of sarcopenia was 21.8% (95% CI: 15.6 to 28.7%, *k* = 2, *I*^2^ = 96.50%, *p* = 0.000). The prevalence in the 2 Mexico studies (29, 47) was 25.4% (95% CI: 21.9 to 28.9%, *k* = 2, *I*^2^ = 95%, *p* = 0.000). Of the three studies conducted in China (31, 33, 46), the pooled prevalence of sarcopenia was 38.6% (95% CI: 28.4 to 49.3%, *k* = 3, *I*^2^ = 82.1%, *p* = 0.004). Among the 2 studies conducted in Turkey (26, 32), the pooled prevalence of sarcopenia was 18.2% (95% CI: 14.7 to 21.9%, *k* = 2, *I*^2^ = 81.20%, *p* = 0.000). The sarcopenia prevalence for the Netherlands (45), Germany (52), and South Korea (42) was 72.6 (95% CI: 66.2 to 78.4%), 75.0 (95% CI: 56.6 to 88.5%), and 24.0 (95% CI: 16.7 to 32.6%) respectively, as reported by one study in each subgroup (Table 3 and Supplementary Figure 2). There were significant between-group differences for subgroup analysis by country for prevalence of sarcopenia (*p* = 0.000). Table 3 and Supplementary Figure 3 also show the pooled prevalence of sarcopenia with geographical area level (Europe vs. Asia vs. North America; pooled prevalence = 57.1% and 30.4 and 25.4%, 95% CI: 26.6 to 84.9% and 19.6 to 42.4% and 21.8 to 28.9%, *k* = 13 and 6 and 2, respectively). There were not significant between-group differences for subgroup analysis by geographical area for prevalence of sarcopenia (*p* = 0.093).

#### Study Population

To identify the prevalence of sarcopenia in different target population, we synthesized the pooled prevalence of sarcopenia stratified by hospitalization status. We only included fifteen cohort studies to perform subgroup analysis here to minimize the risk of bias caused by study design (25–27, 29–31, 41–43, 45–47, 49, 51, 52). Table 3 stratified the analysis according to the three main population settings: (1) ICU patients (only); (2) ICU or COVID-19 nursing ward patients (combined); and (3) COVID-19 nursing ward patients. Subgroup analysis by study population showed significant variation among the subgroups (*p* = 0.000). The prevalence of sarcopenia was more prevalent in ICU patients (69.7%, 95% CI: 51.7 to 85.2%, *k* = 5, *I*^2^ = 91.583%, *p* = 0.000) in comparison with ICU or COVID-19 nursing ward patients (42.0%, 95% CI: 26.5 to 58.3%, *k* = 8, *I*^2^ = 97.10%, *p* = 0.000) and COVID-19 nursing ward patients (28.4%, 95% CI: 22.8 to 34.4%, *k* = 2, *I*^2^ = 60.60%, *p* = 0.111) (Supplementary Figure 4).

#### Study Design

As shown in Table 3, the pooled prevalence for sarcopenia in patients with COVID-19 was 46.4% (95% CI: 27.6 to 65.7%, *k* = 18, *I*^2^ = 99.28%, *p* = 0.000) in the meta-analysis of observational cohort studies and 50.8% (95% CI: 7.1 to 93.7%, *k* = 3, *I*^2^ = 99.8%, *p* = 0.000) in the meta-analysis of cross-sectional studies (Supplementary Figure 5). There were no statistically significant between-group differences in the study design subgroup (*p* = 0.879).

#### Diagnostic Criteria of Sarcopenia

In addition, we performed subgroup meta-analysis of all included studies according to their assessment tools and parameters used to measure sarcopenia. For sarcopenia assessment methods, five studies (26, 30, 33, 42, 47) used chest CT scan with a pooled prevalence of 28% (95% CI: 22.8 to 33.5%, *k* = 5, *I*^2^ = 72.82%, *p* = 0.005), six studies (25, 27, 29, 46, 52, 53) used abdominal CT scan with a pooled prevalence of 63.3% (95% CI: 51.4 to 74.4%, *k* = 6, *I*^2^ = 85.98%, *p* = 0.000), four studies (31, 41, 44, 45) used SARC-F with a pooled prevalence of 73% (95% CI: 49.3 to 91.3%, *k* = 4, *I*^2^ = 98.00%, *p* = 0.000), one study (49) used dynamometer and DXA with a pooled prevalence of 15.8% (95% CI: 10.2 to 23.0%, *k* = 1), and one study (43) used MRC with a pooled prevalence of 69.6% (95% CI: 47.1 to 86.8%, *k* = 1). Other assessment tools included dynamometer (32, 50) and BIA (48, 51), which were used by two studies each and yielded a pooled prevalence of 26.7 (95% CI: 23.2 to 30.5%, *k* = 2, *I*^2^ = 98.90%, *p* = 0.000) and 0.4% (95% CI: 0.1 to 0.8%, *k* = 2, *I*^2^ = 97.70%, *p* = 0.000), respectively. For parameters used to measure sarcopenia, seven studies used skeletal muscle index (SMI) with a pooled prevalence of 49.0% (95% CI: 31.3 to 66.9%, *k* = 7, *I*^2^ = 97.03%, *p* = 0.000) (25, 29, 33, 42, 47, 52, 53), 4 studies used SARF-score with a prevalence of 73% (95% CI: 49.3 to 91.3%, *k* = 4, *I*^2^ = 98.00%, *p* = 0.000) (31, 41, 44, 45), and two studies used HGS with a prevalence of 26.7% (95% CI: 23.2 to 30.5%, *k* = 2, *I*^2^ = 98.90%, *p* = 0.000) (32, 50). The subgroup meta-analysis result of parameters used in the remaining studies is shown in Table 3 and Supplementary Figure 6.

### Sensitivity Analysis and Publication Bias

After deleting a single study, respectively, the results of the pooled prevalence did not materially change, which indicated that the data in our study were relatively credible and stable (Supplementary Figure 7). We also found no evidence of publication bias from the funnel plot (Supplementary Figure 8) and Egger’s test (*p* = 0.000).

## Discussion

To our knowledge, this is the first systematic review and meta-analysis providing an up-to-date estimate of the prevalence of sarcopenia among patients with COVID-19 by combing the data from latest research. Although previous systemic reviews and meta-analyses have investigated the prevalence of sarcopenia in other common geriatric comorbidities (11, 54–56), the prevalence of sarcopenia among patients with COVID-19 has not been widely studied. Our results demonstrated that the overall prevalence of sarcopenia among patients with COVID-19 was 48.0% based on the 21 studies involving 5,407 patients with COVID-19.

Coronavirus disease 2019 is a severe acute infectious disease characterized by a severe inflammatory and highly catabolic status (35). The global pandemic has posed a persisting and unprecedented challenge to global healthcare demand. As ICU patients are at higher risk of COVID-19-related mortality, the initial focus of care was to provide information regarding the clinical characteristics of infection and the affected patients and the associated risk factors with the short-term outcomes to reduce the number of deaths. However, it has become clearer and clearer that survivors of COVID-19, especially in older patients, are at increased risk of acutely developing sarcopenia (23). COVID-19 infection can aggravate acute sarcopenia for several reasons, including the increased muscle wasting provoked by the systematic inflammation, the reduced physical activity, and the presence of poor nutritional status caused by anorexia, anosmia, and social isolation. It is worth emphasizing that acute sarcopenia augments patients’ vulnerability to stressors (57) and may largely have negative consequences on patients’ adverse outcomes during admission as well as persistent decline in the functional and physical abilities in post-COVID-19 condition. Special attention should be paid on the early detection of patients at high risk of sarcopenia, and helping clinicians advance the timing of intervention and propose the most appropriate treatment strategies to avoid the functional and physical deterioration of the patients.

The results from our study showed that sarcopenia is frequently observed in patients with COVID-19. Interestingly, the prevalence of sarcopenia varied significantly among different populations. We found that patients admitted to the ICU had much higher rate of sarcopenia, which is estimated to be 69.7%. This is consistent with previous studies which reported that the prevalence of sarcopenia in critically ill patients was 60–70% (18, 58, 59). Patient admitted to general COVID-19 nursing wards had relatively low rate of sarcopenia, which is estimated to be 28.4%. This result is also consistent with the previous studies reporting only 5–25% of patients admitted to general medical and surgical floors presented with the combination of low muscle mass and strength (60–62). It should also be noted that different countries and screening tools to identify sarcopenia differed significantly. Since different countries apply different diagnostic criteria in the identification, we consider that differences in countries and diagnostic criteria may influence the heterogeneity of the study. Additionally, our subgroup analyses by gender showed that there was no significant difference between the prevalence of sarcopenia in male and female patients, indicating that both male and female patients are vulnerable to develop sarcopenia during COVID-19 infection.

These data on the prevalence of sarcopenia in patients with COVID-19 can also be examined in comparison with that of LSMM prevalence in critically ill patients and patients with COVID-19. Because of its accuracy in body composition measurement and availability in the clinical setting, LSMM is frequently measured through computed tomography scan (CT scan) in clinical practice and is a surrogate parameter for sarcopenia (63, 64). It is observed in a published systematic review and meta-analysis that CT-defined LSMM is highly prevalent in critically ill patients with different underlying diagnoses and the pooled prevalence of LSMM was 50.9% (65). Our results found that sarcopenia is very frequent in critically ill patients with COVID-19 as well, and the prevalence of sarcopenia in critically ill patients with COVID-19 was higher than those with non-critically ill hospitalized patients. In addition, a prior meta-analysis of six studies involving 976 patients with COVID-19 has shown that there were 648 patients with no LSMM (66.4%) and 328 patients with LSMM (33.6%) (28). In this meta-analysis, we synthesized the updated scientific literature evidence and provided a pooled prevalence of sarcopenia for patients with COVID-19 to be 48.0%. The difference between the two studies may be due to the different included articles, differences in assessment tools, and differences in patient populations.

The result of high prevalence of sarcopenia among patients with COVID-19 may not be surprising, because many factors would intensify the acute sarcopenia process during the pandemic. We were more interested in whether diagnostic parameters of sarcopenia could be the predictor of clinical outcomes after adjusting other confounders. The majority of the studies found that CT-derived body composition parameters are linked to poorer outcomes in patients with COVID-19, which is in line with recent meta-analysis findings, indicating that sarcopenia was associated with increased severity and mortality from COVID-19 (28, 34, 66). Ufuk et al. (26) reported that pectoralis muscle area (PMA) and index (PMI) values on chest CT were significantly associated with several adverse outcomes, such as intubation, prolonged hospital stay, and death. Damanti et al.’s (25) CT analyses explored L1, L2, or L3, associating low SMI with negative clinical outcome, such as extubation (OR = 1.02, 95% CI: 1.00–1.03, *p* = 0.017), ICU stay (OR = 0.97, 95% CI: 0.95–0.99, *p* = 0.03), and hospital mortality (hazard ratio = 0.98, 95% CI: 0.96–0.99, *p* = 0.02). McGovern et al. (27) highlighted that 30-day mortality was associated with low SMI (*p* < 0.05) at the level of L3. Osuna-Padilla et al. (29) showed that patients with low muscle mass (defined by SMI on L3 level) had a significantly higher rate of tracheostomy (50 vs. 20%, *p* = 0.01), prolonged ICU (adjusted HR = 0.53, 95%CI: 0.30–0.92, *p* = 0.024), and hospital LOS (adjusted HR = 0.50, 95% CI: 0.29–0.86, *p* = 0.014). Another study by Kim et al. (42) also showed that baseline sarcopenia (defined by SMI on T12 level) was an independent predictor of delayed hospital discharge (adjusted hazard ratio = 0.47; 95% CI 0.23–0.96). Giraudo et al. (30) showed that reduced muscle mass (defined as Hu value < 30) is a predictor of ICU admission. According to Kara et al. (32), those with severe disease had poorer grip strength (26.5 kg/f ± 12.4) than patients with moderate (34.7 kg/f ± 11.1) or mild disease (35.1 kg/f ± 11.2). However, some present studies have also found no significant association between body composition parameters and adverse outcomes, such as disease severity, systemic inflammation, length of stay, IMV, or time to death (47, 48, 52, 53). Overall, these findings appear that body composition may play an essential role in predicting clinical outcome in patients with COVID-19. More large-scale studies are needed to determine the prognostic role of body composition in these patients.

### Clinical Practice

According to our evidence, our study highlights the importance of considering the risk of acute sarcopenia in patients with COVID-19 during hospital stay and after discharge. Since scientific community did not pay much attention in studying the long-term evolution on muscle performance of the survivors of COVID-19 previously, measurement of muscle strength or muscle quantity has not been used in clinical care of patients with COVID-19 routinely. Besides, as the precise diagnose of sarcopenia is not easy to be identified, acute sarcopenia may go unnoticed until it goes an extremely serious state. Hence, we suggest that integration of serial measurements of muscle strength, physical performance, and muscle quantity should be conducted by clinicians in clinical practice, allowing them to timely detect patients with high risk of sarcopenia and forge a dynamic intervention plan when there is a change.

### Limitations

Our results should be interpreted in the light of some potential limitations. First, our study had a relatively small sample size, and the majority of patient population were limited to those in hospitalization. A recent study reported the prevalence of sarcopenia in the community-dwelling oldest-old population during the pandemic is high, with an estimated prevalence of 24.5% (67). This calls for more multi-site large-scale cohort studies involving community-dwelling residents and patients recruited from the hospital-based system to provide a more complete picture of the muscle impact of COVID-19. Second, some other potential risk factors may contribute for the nutritional or muscle status, such as lifestyle habits, chronic disease, and physical activity. But these factors were not addressed in this study. Thus, the distinguishment of baseline muscle characteristics might be of particular importance to define whether sarcopenia was caused before or during COVID-19 infection. Third, notable heterogeneities were identified in this study. This could be owing to the fact that the included studies used different types of sarcopenia assessment tools, parameters measured of different thoracic levels or lumbar levels, and/or different muscles, and various cutoff values. Given that SMI at T12 seems to have a lower correlation with total body muscle mass than SMI at L3 (68), skeletal muscle mass should be assessed on the level L3 using validated cutoffs and it should include all muscles. Third, owing to the COVID-19 restriction, some included studies estimated sarcopenia risk using the SARC-F scale, which is a rudimentary assessment based on self-reported data; therefore, recall bias was unavoidable. Fourth, the presence of sarcopenia was defined only based on muscle mass in the majority of studies, whereas sarcopenia is more recognized based on the evaluation of other quantitative (e.g., grip strength, DXA) and qualitative tests (e.g., Chair stand test, 400-m walk test). However, applying these tests during a pandemic is extremely difficult. Fifth, publication limitation could have been present due to the inclusion of English-only published studies. Finally, since the focus of this study is to provide information concerning the estimated prevalence of sarcopenia in COVID-19, and not so much in studying the prognostic value of sarcopenia. To establish the probable association between sarcopenia and clinical outcomes, more research with longitudinal tracking of prognostic outcomes with sarcopenia in COVID-19 survivors is urgently warranted.

## Conclusion

Our findings suggested that 48.0% of patients with COVID-19 are at high risk of developing sarcopenia, which highlights the importance to screen and diagnose sarcopenia. In addition, sarcopenia is frequently observed in patients with COVID-19, with varying prevalence depending on study countries, hospitalization status, and measurement tools used. Patients with sarcopenic risk should be monitored more carefully when hospitalized during COVID-19.

## Data Availability Statement

The raw data supporting the conclusion of this article will be made available by the authors, without undue reservation.

## Author Contributions

YX contributed to conception, design, and drafting of the manuscript. YX, J-WX, PY, B-LW, and CL contributed to acquisition, analysis, or interpretation of data. YX and J-WX contributed to statistical analysis. T-HT and C-WC contributed to supervision. All authors have read and agreed to the published version of the manuscript.

## Conflict of Interest

The authors declare that the research was conducted in the absence of any commercial or financial relationships that could be construed as a potential conflict of interest.

## Publisher’s Note

All claims expressed in this article are solely those of the authors and do not necessarily represent those of their affiliated organizations, or those of the publisher, the editors and the reviewers. Any product that may be evaluated in this article, or claim that may be made by its manufacturer, is not guaranteed or endorsed by the publisher.
