# Supplementary material for: Prevalence of Sarcopenia in Patients With COVID-19: A Systematic Review and Meta-Analysis
Source: Front Nutr. 2022 Jul 4;9:925606. doi: 10.3389/fnut.2022.925606 (PMC9289534; doi:10.3389/fnut.2022.925606)
Supplement: Supplementary file 1 [file Data_Sheet_1.docx]

**Frontiers in nutrition**

**Prevalence of sarcopenia in COVID-19 patients: a systematic review and meta-analysis | Supplementary Materials**

| **Table S1** | PRISMA Checklist. | p.2 |  |
| --- | --- | --- | --- |
| **Table S2** | PECOS criteria for inclusion in the systematic review and meta-analysis. | p.4 |  |
| **Table S3** | Search Strategy. | p.5 |  |
| **Table S4** | Quality assessment of observational studies using Newcastle-Ottawa Scales. | p.8 |  |
| **Table S5** | Quality assessment of cross-sectional studies using Joanna Briggs Institute manual. | p.9 |  |
| **Figure S1** | Subgroup Analysis by Sex, Sarcopenia Prevalence. | p.10 |  |
| **Figure S2** | Subgroup Analysis by Study Countries, Sarcopenia Prevalence. | p.12 |  |
| **Figure S3** | Subgroup Analysis by Study Regions, Sarcopenia Prevalence. | p.13 |  |
| **Figure S4** | Subgroup Analysis by Study Population, Sarcopenia Prevalence. | p.14 |  |
| **Figure S5** | Subgroup Analysis by Study Design, Sarcopenia Prevalence. | p.15 |  |
| **Figure S6** | Subgroup Analysis by Diagnostic criteria, Sarcopenia Prevalence. | p.16 |  |
| **Figure S7** | Sensitivity Analysis, Sarcopenia Prevalence. | p.18 |  |
| **Figure S8** | Funnel Plot, Sarcopenia Prevalence. | p.19 |  |

**Table S1** PRISMA Checklist

| **Section/topic** | |  | **#** | **Checklist item** | **Reported on page #** |
| --- | --- | --- | --- | --- | --- |
|  | **TITLE** | | | | |
| Title | |  | 1 | Identify the report as a systematic review, meta-analysis, or both. | 1 |
|  | **ABSTRACT** | | | |  |
| Structured summary | |  | 2 | Provide a structured summary including, as applicable: background; objectives; data sources; study eligibility criteria, participants, and interventions; study appraisal and synthesis methods; results; limitations; conclusions and implications of key findings; systematic review registration number. | 2-3 |
|  | **INTRODUCTION** | | | |  |
| Rationale | |  | 3 | Describe the rationale for the review in the context of what is already known. | 4-6 |
| Objectives | |  | 4 | Provide an explicit statement of questions being addressed with reference to participants, interventions, comparisons, outcomes, and study design (PICOS). | 6-7 |
|  | **METHODS** | | | |  |
| Protocol and registration | |  | 5 | Indicate if a review protocol exists, if and where it can be accessed (e.g., Web address), and, if available, provide registration information including registration number. | 7 |
| Eligibility criteria | |  | 6 | Specify study characteristics (e.g., PICOS, length of follow-up) and report characteristics (e.g., years considered, language, publication status) used as criteria for eligibility, giving rationale. | 7 |
| Information sources | |  | 7 | Describe all information sources (e.g., databases with dates of coverage, contact with study authors to identify additional studies) in the search and date last searched. | 6 |
| Search | |  | 8 | Present full electronic search strategy for at least one database, including any limits used, such that it could be repeated. | Table S3 |
| Study selection | |  | 9 | State the process for selecting studies (i.e., screening, eligibility, included in systematic review, and, if applicable, included in the meta-analysis). | 7 |
| Data collection process | |  | 10 | Describe method of data extraction from reports (e.g., piloted forms, independently, in duplicate) and any processes for obtaining and confirming data from investigators. | 8 |
| Data items | |  | 11 | List and define all variables for which data were sought (e.g., PICOS, funding sources) and any assumptions and simplifications made. | 8 |
| Risk of bias in individual studies | |  | 12 | Describe methods used for assessing risk of bias of individual studies (including specification of whether this was done at the study or outcome level), and how this information is to be used in any data synthesis. | 8 |
| Summary measures | |  | 13 | State the principal summary measures (e.g., risk ratio, difference in means). | 9 |
| Synthesis of results | |  | 14 | Describe the methods of handling data and combining results of studies, if done, including measures of consistency (e.g., I^2^) for each meta-analysis. | 9 |
| Risk of bias across studies | |  | 15 | Specify any assessment of risk of bias that may affect the cumulative evidence (e.g., publication bias, selective reporting within studies). | 9 |
| Additional analyses | |  | 16 | Describe methods of additional analyses (e.g., sensitivity or subgroup analyses, meta-regression), if done, indicating which were pre-specified. | 9 |
|  | **RESULTS** | | | |  |
| Study selection | |  | 17 | Give numbers of studies screened, assessed for eligibility, and included in the review, with reasons for exclusions at each stage, ideally with a flow diagram. | Fig. 1. |
| Study characteristics | |  | 18 | For each study, present characteristics for which data were extracted (e.g., study size, PICOS, follow-up period) and provide the citations. | 10-11 |
| Risk of bias within studies | |  | 19 | Present data on risk of bias of each study and, if available, any outcome level assessment (see item 12). | Table S4, Table S5 |
| Results of individual studies | |  | 20 | For all outcomes considered (benefits or harms), present, for each study: (a) simple summary data for each intervention group (b) effect estimates and confidence intervals, ideally with a forest plot. | 9-12  Fig. 2 |
| Synthesis of results | |  | 21 | Present results of each meta-analysis done, including confidence intervals and measures of consistency. | 9-16 |
| Risk of bias across studies | |  | 22 | Present results of any assessment of risk of bias across studies (see Item 15). | 11-12 |
| Additional analysis | |  | 23 | Give results of additional analyses, if done (e.g., sensitivity or subgroup analyses, meta-regression [see Item 16]). | 12-16 |
|  | **DISCUSSION** | | | |  |
| Summary of evidence | |  | 24 | Summarize the main findings including the strength of evidence for each main outcome; consider their relevance to key groups (e.g., healthcare providers, users, and policy makers). | 16-21 |
| Limitations | |  | 25 | Discuss limitations at study and outcome level (e.g., risk of bias), and at review-level (e.g., incomplete retrieval of identified research, reporting bias). | 21-23 |
| Conclusions | |  | 26 | Provide a general interpretation of the results in the context of other evidence, and implications for future research. | 24 |
|  | **FUNDING** | | | |  |
| Funding | |  | 27 | Describe sources of funding for the systematic review and other support (e.g., supply of data); role of funders for the systematic review. | N/A |

**Table S2** PECOS criteria for inclusion in the systematic review and meta-analysis.

| **Parameter** | **Inclusion criteria** |
| --- | --- |
| Population | General population or hospitalized population |
| Exposure | The exposures of interest are infection with COVID-19 |
| Comparison/comparator | The comparator will be healthy population that without COVID-19 |
| Outcomes | The outcome of interest is diagnosis of sarcopenia |
| Study design | Cohort studies, case-control, and cross-sectional studies |

**Table S3** Search Strategy

| Source | Search Strategies |
| --- | --- |
| **Pubmed**  **(N=415)** | #1 (coronavirus infections[MeSH Terms]) OR (coronavirus[Title/Abstract] OR covid-19[Title/Abstract] OR SARS-CoV-2[Title/Abstract] OR Severe Acute Respiratory Syndrome[Title/Abstract] OR 2019-nCoV[Title/Abstract] OR 2019nCoV[Title/Abstract]  n=226867  #2 (sarcopenia OR Muscular Atrophy OR Muscle Weakness[MeSH Terms]) OR (muscle loss[Title/Abstract] OR muscle depletion[Title/Abstract] OR muscle reduction[Title/Abstract] OR muscle wasting[Title/Abstract] OR reduced muscle[Title/Abstract] OR loss of muscle[Title/Abstract] OR muscle mass[Title/Abstract] OR muscle atrophy[Title/Abstract] OR muscular weakness[Title/Abstract]) n=78226  #3 #1 AND #2 n=280  Search date:2022/01/09  #1 (coronavirus infections[MeSH Terms]) OR coronavirus[Title/Abstract] OR covid-19[Title/Abstract] OR SARS-CoV-2[Title/Abstract] OR Severe Acute Respiratory Syndrome[Title/Abstract] OR 2019-nCoV[Title/Abstract] OR 2019nCoV[Title/Abstract]  n=272744  #2 ("Low muscle mass"[Title/Abstract]) OR ("Body composition"[Title/Abstract]) n=43414  #3 #1 AND #2 n=135  Search date:2022/05/19 |
| **Embase**  **(N=1176)** | #1 'sarcopenia'/exp OR 'muscle atrophy'/exp OR 'muscle weakness'/exp OR 'muscle loss':ab,ti OR 'muscle depletion':ab,ti OR 'muscle reduction':ab,ti OR 'muscle wasting':ab,ti OR 'reduced muscle':ab,ti OR 'loss of muscle':ab,ti OR 'muscle mass':ab,ti OR 'muscular atrophy':ab,ti OR 'muscular weakness':ab,ti n=486636  #2 'coronavirus infection'/exp OR coronavirus:ab,ti OR 'covid 19':ab,ti OR 'sars cov 2':ab,ti OR '2019 ncov':ab,ti OR 2019ncov:ab,ti OR 'severe acute respiratory syndrome':ab,ti  n=241945  #3 #1 AND #2 n=2524  #4 #3 AND 'human'/de AND 'article'/it n=1081  Search date:2022/01/09  #1 'coronavirus infection'/exp OR 'coronavirus infection' OR coronavirus:ab,ti OR 'covid 19':ab,ti OR 'sars cov 2':ab,ti OR '2019 ncov':ab,ti OR 2019ncov:ab,ti OR 'severe acute respiratory syndrome':ab,ti n=301912  #2 'low muscle mass':ab,ti OR 'body composition':ab,ti n=58456  #3 #1 AND #2 n=165  #4 #3 AND 'article'/it n=95  Search date:2022/05/19 |
| **Web of Science**  **(N=1567)** | #1 TS=(Sarcopenia OR muscle loss OR muscle depletion OR muscle reduction OR muscle wasting OR reduced muscle OR loss of muscle OR muscle mass OR muscle atrophy OR OR Muscular Atrophy OR muscle weakness OR muscular weakness)  n=653808  #2 TS=(coronavirus OR covid-19 OR SARS-CoV-2 OR coronavirus infections OR Severe Acute Respiratory Syndrome OR 2019-nCoV OR 2019nCoV)  n=389850  #3 #1 AND #2 n=1847  #4 TS=(mouse OR rat OR mice) n=6365850  #5 #3 NOT #4 n=1730  #6 #5 and Articles (Document Types) n=1360  Search date:2022/01/09  #1 TS = (coronavirus OR covid-19 OR SARS-CoV-2 OR coronavirus infections OR Severe Acute Respiratory Syndrome OR 2019-nCoV OR 2019nCoV) n=474922  #2 TS=(“Low muscle mass” OR “Body composition”) n=130506  #3 (TS=(“Low muscle mass” OR “Body composition”)) AND TS=( coronavirus OR covid-19 OR SARS-CoV-2 OR coronavirus infections OR Severe Acute Respiratory Syndrome OR 2019-nCoV OR 2019nCoV ) n=244  #4 TS=(mouse OR rat OR mice) n=6443188  #5 #3 NOT #4 n=236  #6 #3 NOT #4 and Article or Clinical Trial (Document Types) n=207  Search date:2022/05/19 |
| **The Cochrane library**  **(N=261)** | #1 MeSH descriptor: [Sarcopenia] explode all trees n=560  #2 MeSH descriptor: [Muscular Atrophy] explode all trees n=912  #3 MeSH descriptor: [Muscle Weakness] explode all trees n=583  #4 (muscle loss OR muscle depletion OR muscle reduction OR muscle wasting OR reduced muscle OR loss of muscle OR muscle mass OR muscle atrophy OR muscular weakness):ti,ab,kw n=36800  #5 #1 OR #2 OR #3 OR #4 n=37343  #6 MeSH descriptor: [Coronavirus Infections] explode all trees n=1463  #7 (coronavirus OR covid-19 OR SARS-CoV-2 OR Severe Acute Respiratory Syndrome OR 2019nCoV) n=9870  #8 # 6 OR #7 n=9870  #9 #5 AND #8 in trials n=220  Search date:2022/01/09  #1 "Low muscle mass" OR "body composition" n=15172  #2 (coronavirus OR covid-19 OR SARS-CoV-2 OR Severe Acute Respiratory Syndrome OR 2019nCoV) n=12578  #3 #1 AND #2 n=72  #4 #1 AND #2 in trials n=41  Search date:2022/05/19 |
| **Scopus**  **(N=1220)** | ( TITLE-ABS-KEY ( "coronavirus infections" OR "coronavirus" OR "covid-19" OR "SARS-CoV-2" OR "Severe Acute Respiratory Syndrome" OR "2019-nCoV" OR "2019nCoV" ) AND TITLE-ABS-KEY ( "sarcopenia" OR "Muscular Atrophy" OR "Muscle Weakness" OR "muscle loss" OR "muscle depletion" OR "muscle reduction" OR "muscle wasting" OR "reduced muscle" OR "loss of muscle" OR "muscle mass" OR "muscle atrophy" OR "muscular weakness" OR "Low muscle mass" OR "Body composition" ) ) n=1220  Search date:2022/05/19 |

**Table S4** Quality assessment of observational studies using Newcastle-Ottawa Scales.

| Author | Year | Selection |  |  |  | Comparability | Outcome | |  | |  | Quality (9 point) |
| --- | --- | --- | --- | --- | --- | --- | --- | --- | --- | --- | --- | --- |
|  |  | Representativeness of the exposed cohort (1 point) | Selection of the non-exposed cohort (1 point) | Ascertainment of exposure (1 point) | Demonstration that outcome of interest was not present at start of study (1 point) | Comparability of cohorts on the basis of the design or analysis controlled for confounders (2 point) | Assessment of outcome (1 point) | Was follow-up long enough for outcomes to occur (1 point) | | Adequacy of follow-up of cohorts (1 point) | |  |
| Ufuk | 2020 | 1 | 1 | 1 | 1 | 2 | 1 | | 1 | | 1 | 9 |
| Yang | 2020 | 1 | 1 | 1 | 0 | 2 | 1 | | 1 | | 1 | 8 |
| Cuerda | 2021 | 1 | 1 | 1 | 1 | 0 | 1 | | 1 | | 1 | 7 |
| Damanti | 2021 | 1 | 1 | 1 | 1 | 2 | 1 | | 1 | | 1 | 9 |
| Giraudo | 2021 | 1 | 1 | 1 | 1 | 0 | 1 | | 1 | | 1 | 7 |
| Kim | 2021 | 1 | 1 | 1 | 1 | 2 | 1 | | 1 | | 1 | 9 |
| Ma | 2021 | 1 | 1 | 1 | 1 | 2 | 1 | | 1 | | 1 | 9 |
| Medrinal | 2021 | 1 | 1 | 1 | 0 | 0 | 1 | | 1 | | 1 | 6 |
| Wierdsma | 2021 | 1 | 1 | 1 | 0 | 0 | 1 | | 1 | | 1 | 6 |
| McGovern J | 2021 | 1 | 1 | 1 | 1 | 0 | 1 | | 1 | | 1 | 7 |
| Moctezuma-Velazquez | 2021 | 1 | 1 | 1 | 1 | 2 | 1 | | 1 | | 1 | 9 |
| Yi X | 2021 | 1 | 1 | 1 | 1 | 0 | 1 | | 1 | | 1 | 7 |
| Gobbi | 2021 | 1 | 1 | 1 | 0 | 0 | 1 | | 1 | | 1 | 6 |
| Wilkinson | 2021 | 1 | 1 | 1 | 0 | 2 | 1 | | 1 | | 1 | 8 |
| Osuna-Padilla | 2022 | 1 | 1 | 1 | 1 | 2 | 1 | | 1 | | 1 | 9 |
| Molwitz | 2022 | 1 | 1 | 1 | 1 | 2 | 1 | | 1 | | 1 | 9 |
| Levy | 2022 | 1 | 1 | 1 | 1 | 0 | 1 | | 1 | | 1 | 7 |
| McGovern J | 2022 | 1 | 1 | 1 | 1 | 0 | 1 | | 1 | | 1 | 7 |

**Table S5** Quality assessment of cross-sectional studies using Joanna Briggs Institute manual.

| Joanna Briggs Institute critical appraisal checklist for analytical cross-sectional studies | Reference | | |  |
| --- | --- | --- | --- | --- |
| Criteria | Riesgo 2021 | Kara O 2021 | Damanti 2022 |  |
| 1. Were the criteria for inclusion in the sample clearly defined? | Y | Y | Y |  |
| 2. Were the study subjects and the setting described in detail? | Y | Y | Y |  |
| 3. Was the exposure measured in a valid and reliable way? | Y | Y | Y |  |
| 4. Were objective, standard criteria used for measurement of the condition? | Y | Y | Y |  |
| 5. Were confounding factors identified? | Y | Y | N |  |
| 6. Were strategies to deal with confounding factors stated? | U | U | U |  |
| 7. Were the outcomes measured in a valid and reliable way? | Y | Y | Y |  |
| 8. Was an appropriate statistical analysis used? | Y | Y | Y |  |
| Y: Yes, U: No Unclear, N: Not applicable |  |  |  |  |

**Figure S1** Subgroup Analysis by Sex, Sarcopenia Prevalence

A. Sarcopenia Prevalence in Men


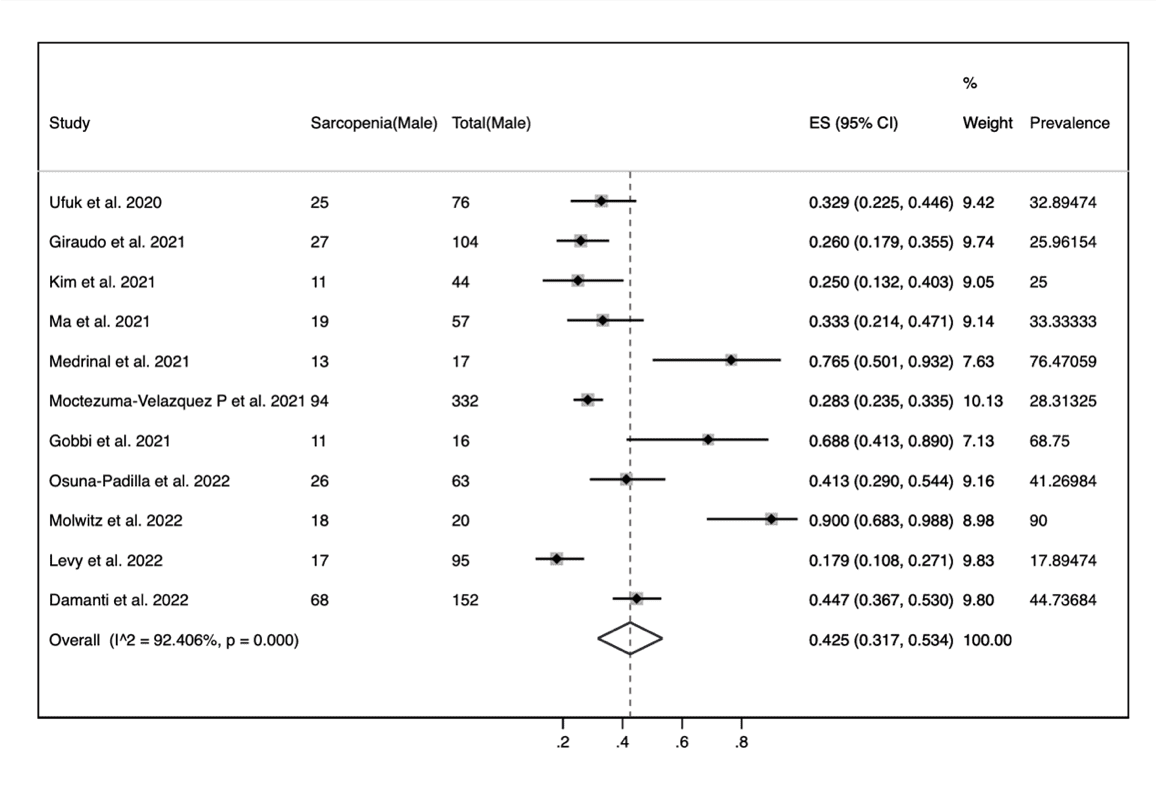


**Figure S1** Subgroup Analysis by Sex, Sarcopenia Prevalence

B. Sarcopenia Prevalence in Women


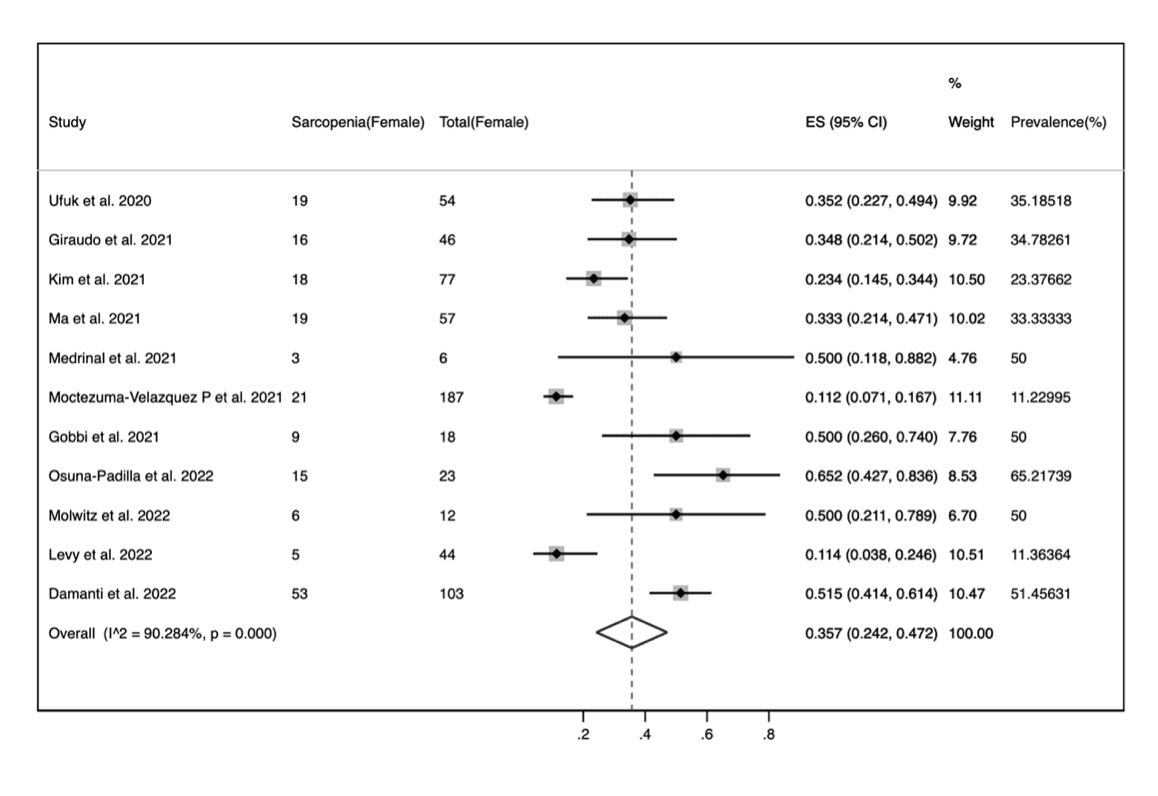


**Figure S2** Subgroup Analysis by Study Countries, Sarcopenia Prevalence


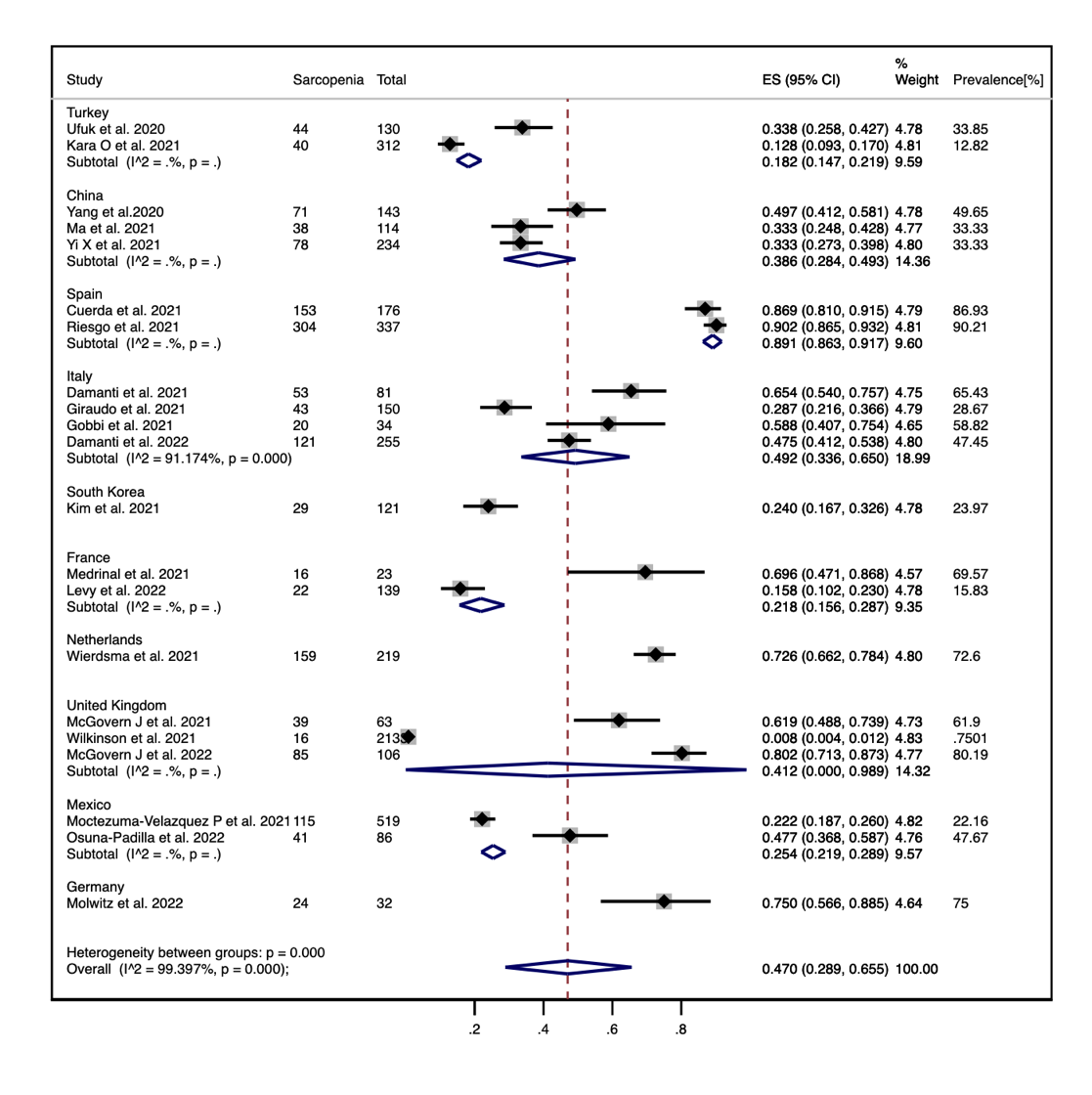


The difference between subgroups was statistically significant (p=0.000).

**Figure S3** Subgroup Analysis by Study Regions, Sarcopenia Prevalence


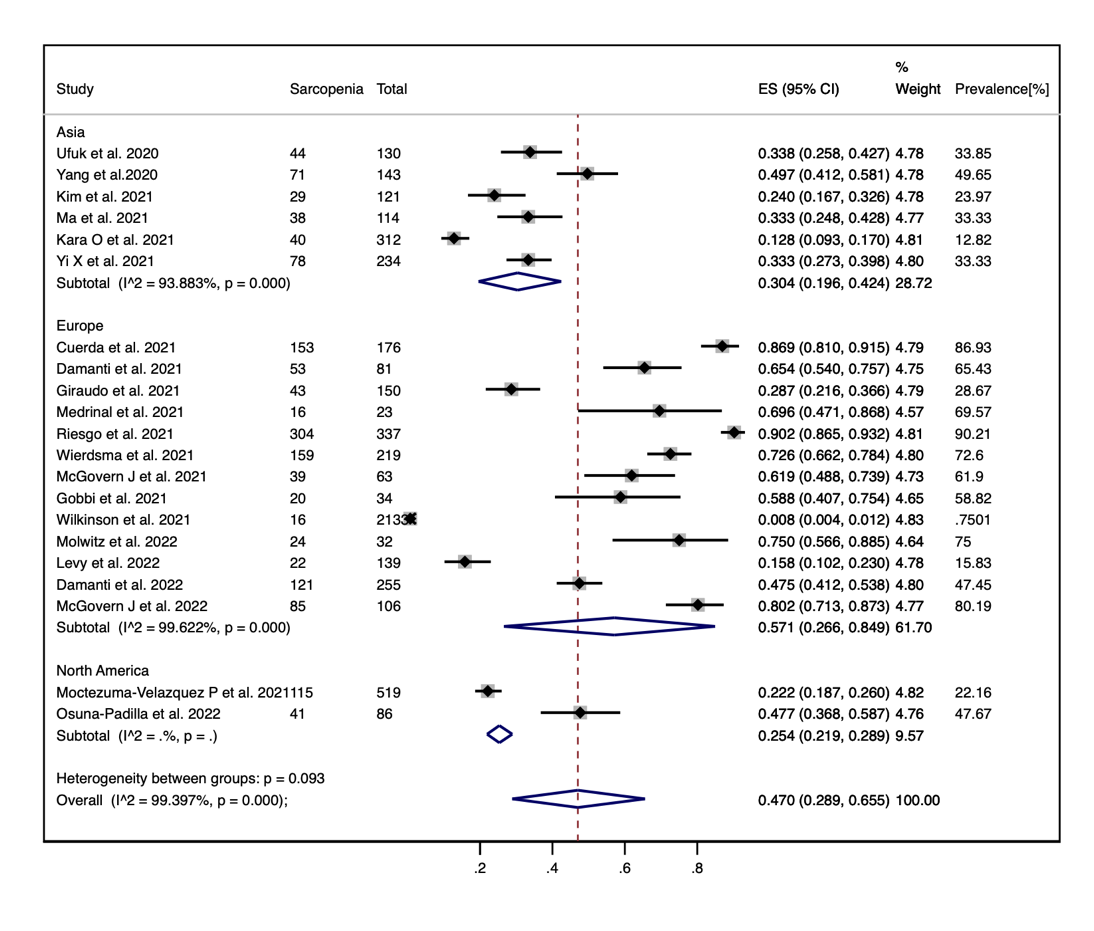


The difference between subgroups was not statistically significant (p=0.093).

**Figure S4** Subgroup Analysis by Study Population, Sarcopenia Prevalence


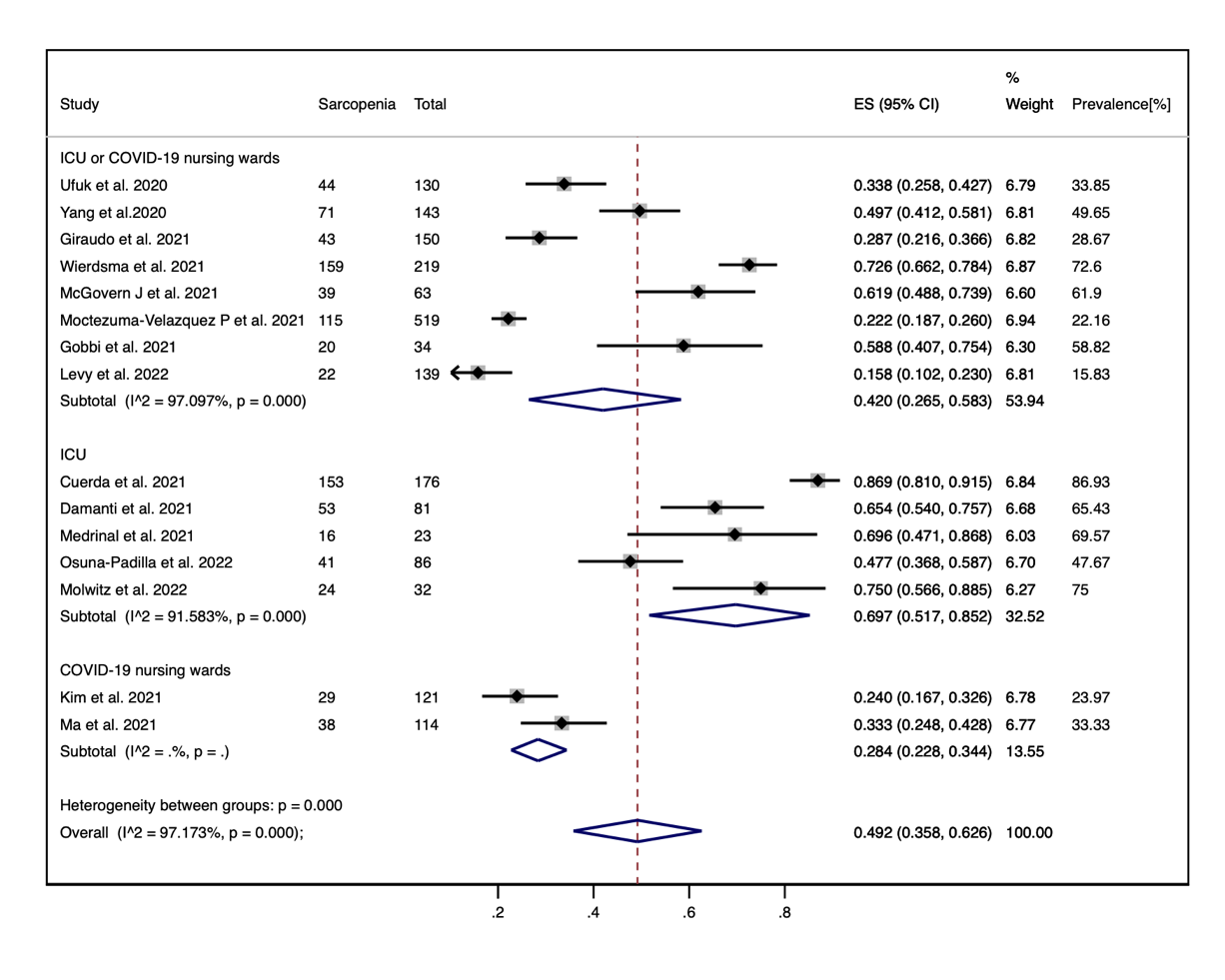


The difference between subgroups was statistically significant (p=0.000).

**Figure S5** Subgroup Analysis by Study Design, Sarcopenia Prevalence


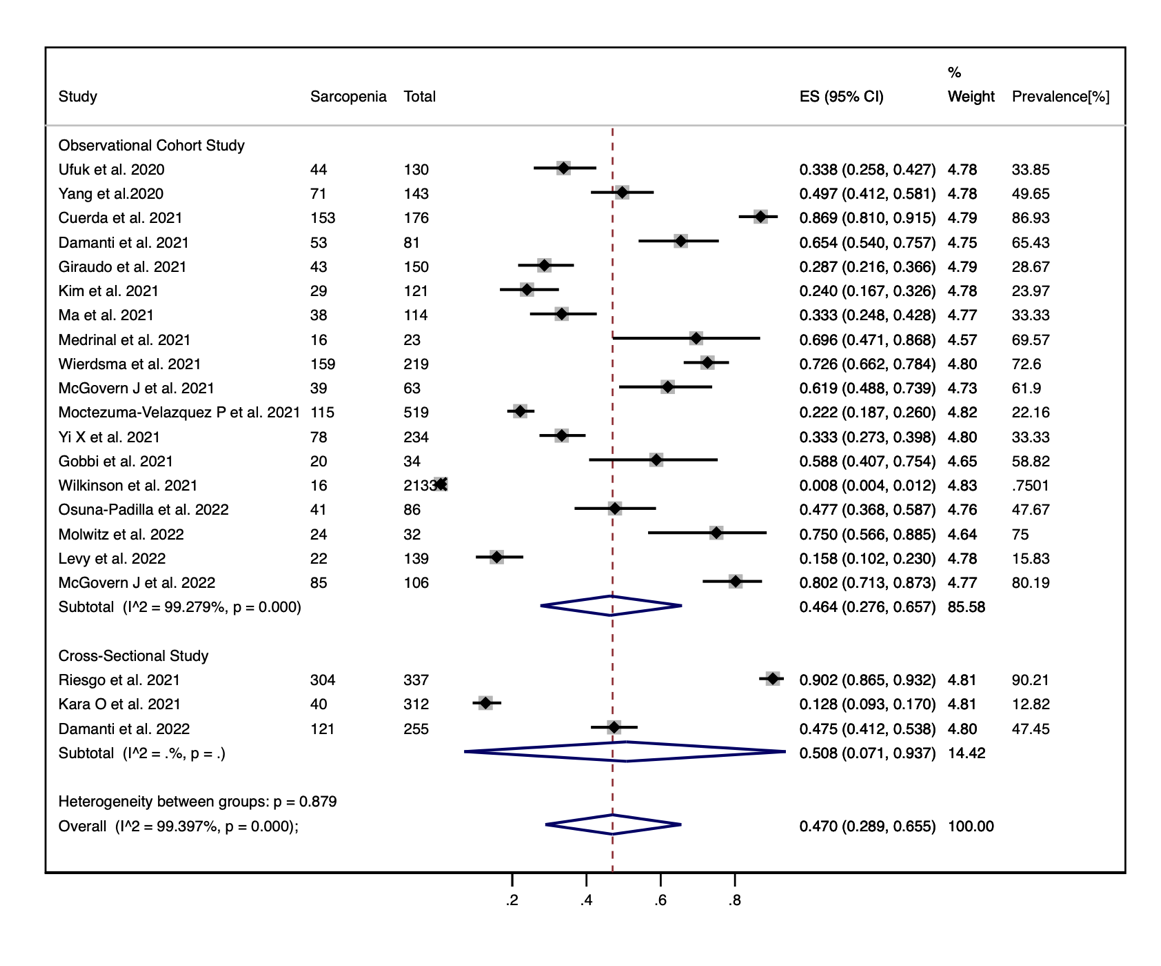


The difference between subgroups was statistically significant (p=0.879).

**Figure S6** Subgroup Analysis by Diagnostic criteria, Sarcopenia Prevalence

1. Assessment tools.


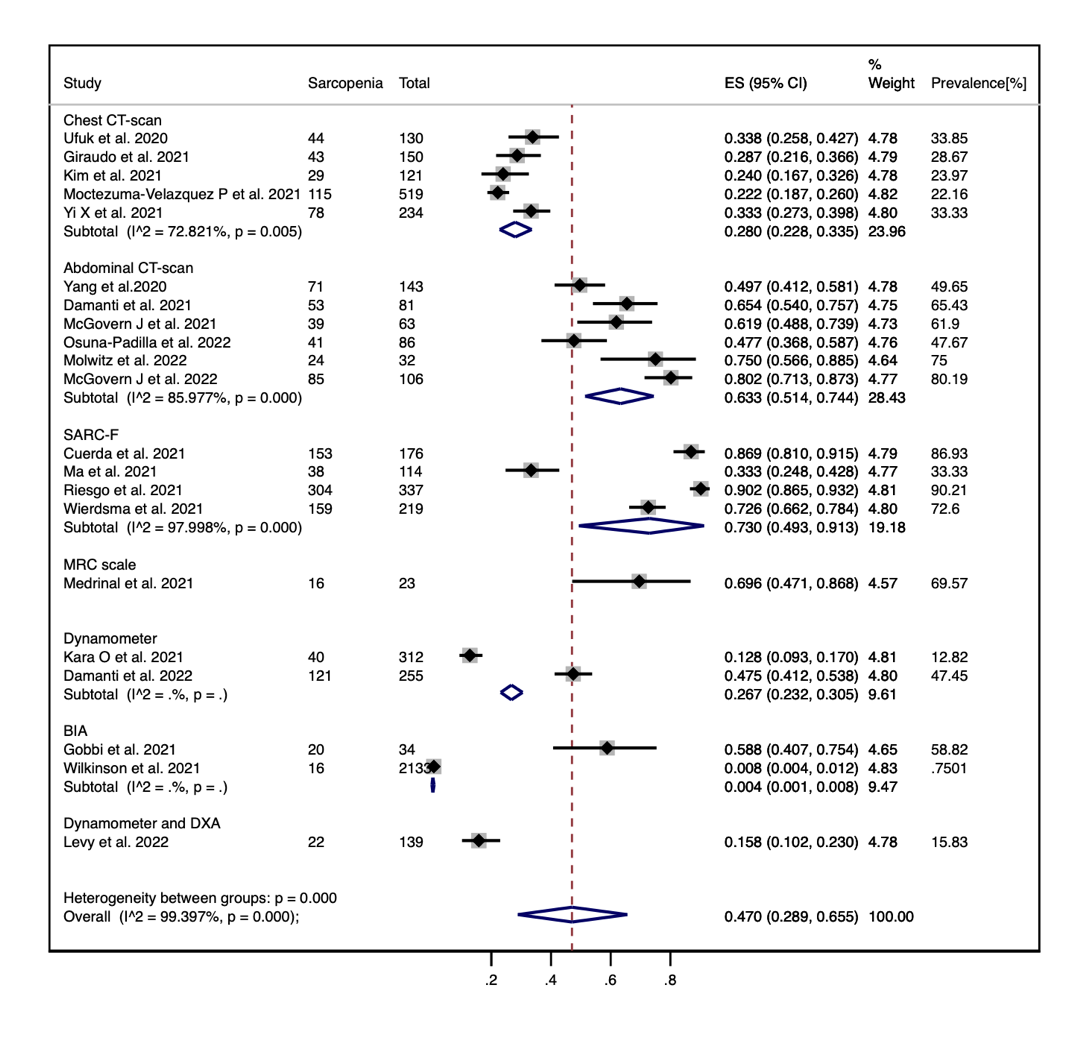


The difference between subgroups was statistically significant (p=0.000).

**Figure S6** Subgroup Analysis by Diagnostic criteria, Sarcopenia Prevalence

1. Parameters used.


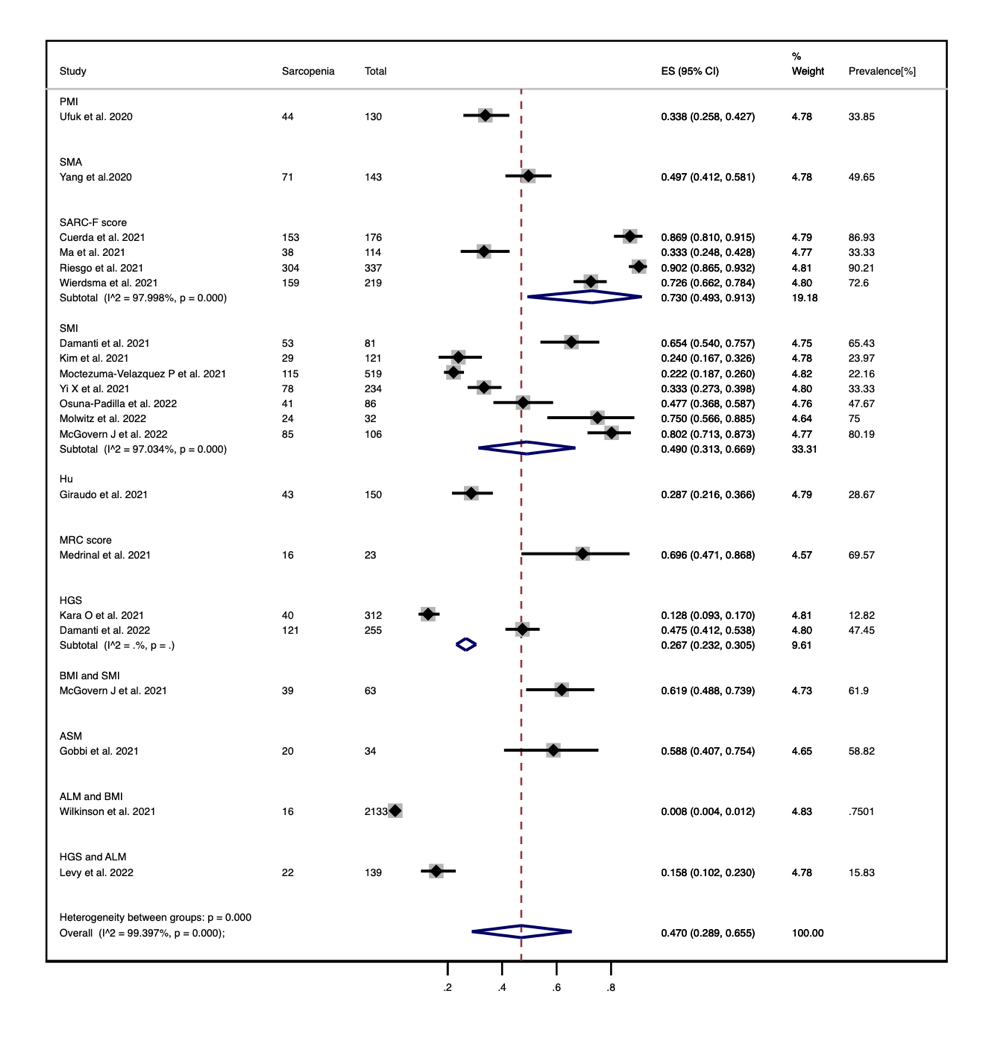


The difference between subgroups was statistically significant (p=0.000).

**Figure S7** Sensitivity Analysis, Sarcopenia Prevalence

**
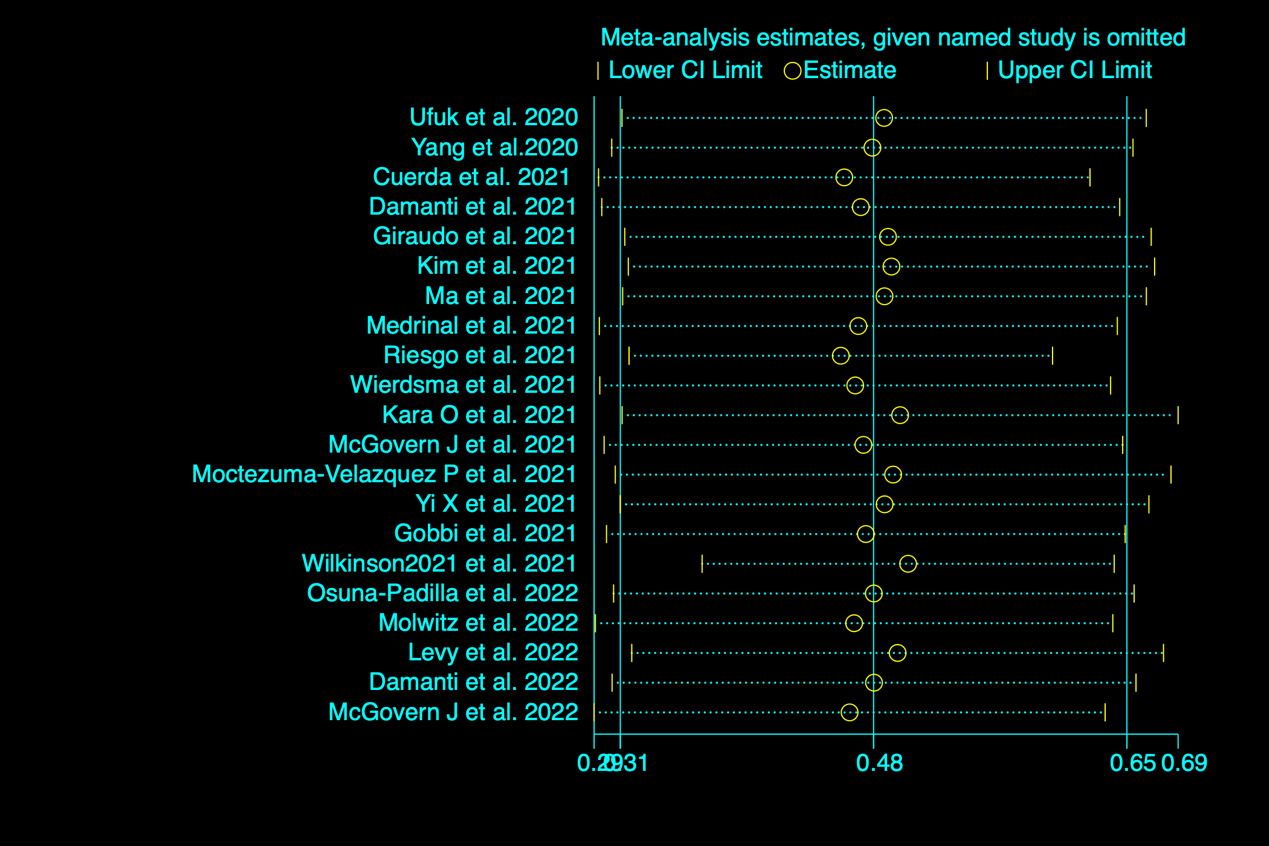
**

**Figure S8** Funnel Plot, Sarcopenia Prevalence


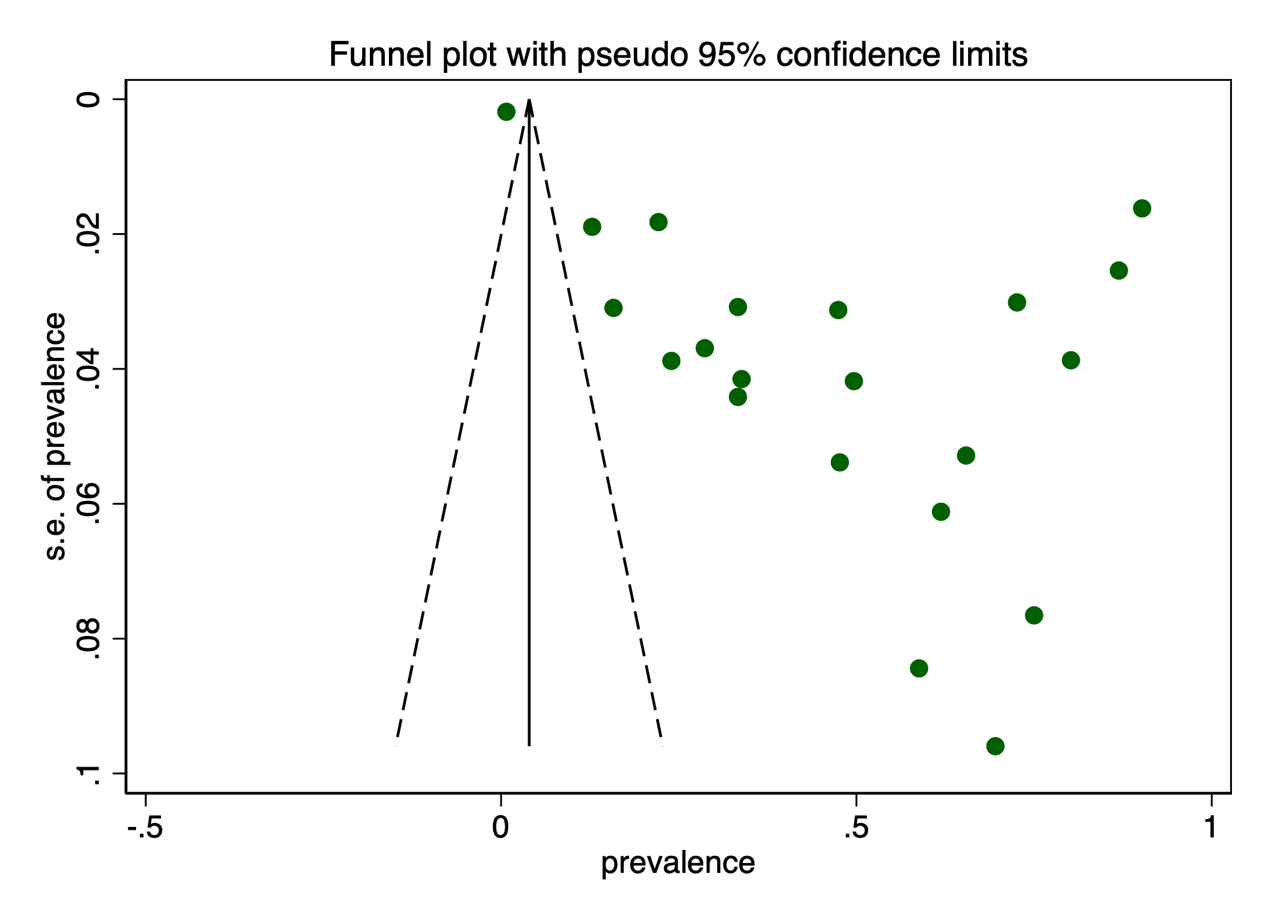


Egger’s test show that there is no publication bias (p=0.000)
